# Molecular basis of vesicular monoamine transport and neurological drug interactions

**DOI:** 10.1016/j.celrep.2025.116490

**Published:** 2025-10-29

**Authors:** Jin Ye, Huaping Chen, Aaron Ammerman, Yi Wang, Kaituo Wang, Jinbin Xu, Bin Liu, Weikai Li

**Affiliations:** 1 Department of Biochemistry and Molecular Biophysics, Washington University School of Medicine, St. Louis, MO 63110, USA; 2 Department of Radiology, Washington University School of Medicine, St. Louis, MO 63110, USA; 3 Department of Biomedical Sciences, University of Copenhagen, Copenhagen, Denmark; 4 The Hormel Institute, University of Minnesota, Austin, MN 55912, USA; 5 Senior author; 6 Lead contact

## Abstract

Vesicular monoamine transporter 2 (VMAT2) stores monoamine neurotransmitters in synaptic vesicles to regulate their release. VMAT2 is a primary target in neurological disorder treatment and contributes to amphetamine-induced psychostimulation. Here, we report cryo-electron microscopy structures of human VMAT2 capturing a cytoplasmic-open state with reserpine and lumenal-facing states with serotonin and histamine in open, amphetamine in less open, tetrabenazine in fully occluded, and unbound VMAT2 in partially occluded conformations. This structural flexibility facilitates tetrabenazine binding and proton-driven monoamine accumulation. VMAT2 binds serotonin and histamine in opposite orientations through two negatively charged sites, one functioning in protonation. Amphetamine binds in the same pocket without engaging this protonation site. Liposome-based analyses demonstrate that amphetamine directly induces the release of a fluorescent monoamine analog via VMAT2, consistent with an exchange mechanism underlying psychostimulation. These findings reveal the molecular basis of monoamine storage and drug interactions by VMAT2, informing therapeutic development for neurological diseases and substance abuse.

## In brief

Ye et al. reveal how VMAT2 loads monoamine neurotransmitters into storage vesicles and interacts with neurological drugs, facilitated by the structural flexibility of the transporter. Amphetamine directly triggers monoamine release to induce psychostimulation, likely by bypassing the regular transport cycle. These insights elucidate psychostimulant action and inform therapeutic strategies.

## Garphical Abstract

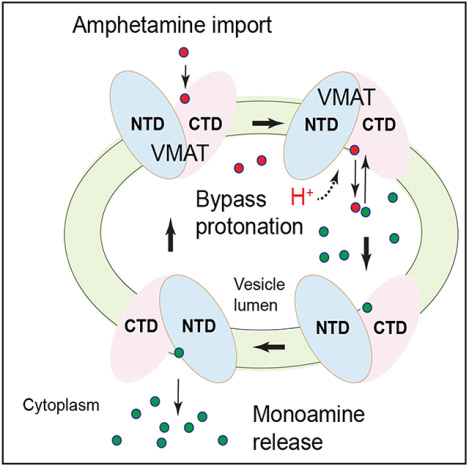

## INTRODUCTION

**Figure 1.**
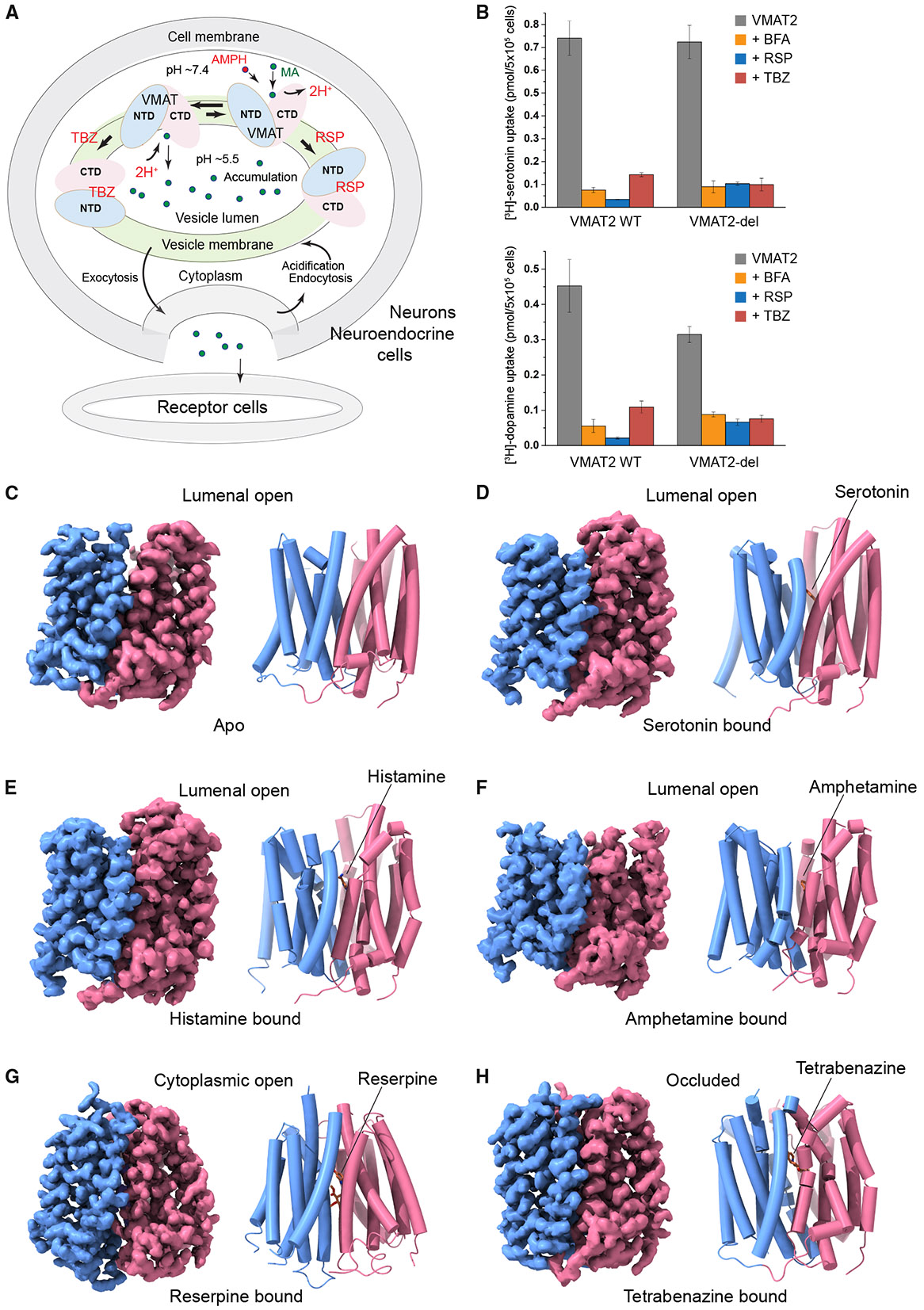
VMAT2 function and overall VMAT2 structures (A) VMAT-mediated monoamine storage, release, and drug interactions in neurons and neuroendocrine cells. VMAT1 and 2 accumulate various monoamines (MA, green spheres) in secretory vesicles via proton (2H^+^) antiport. This process is facilitated by the rocker-switch transition of VMATs, alternating between lumenal-facing and cytoplasmic-facing conformations. Amphetamine (AMPH, red sphere) promotes vesicular monoamine release via VMATs. Reserpine (RSP) and tetrabenazine (TBZ) inhibit VMAT2 in the cytoplasmic-facing and periplasmic-facing conformations, respectively. The N-terminal domain (NTD) and C-terminal domain (CTD) of VMAT2 are colored in blue and red, respectively. (B) VMAT2-del maintains the wild-type (WT) VMAT2 function. Vesicular uptake of [^3^H]-serotonin (top) and [^3^H]-dopamine (bottom) was measured in HEK293T cells after plasma membrane permeabilization. VMAT2-del shows activities similar to WT, and both activities are inhibited by bafilomycin A (BFA), which collapses the proton gradient, and by VMAT2-specific inhibitors, reserpine and tetrabenazine. (C–H) Cryo-EM density maps (left) and structures (right) of VMAT2 in unbound state (C) (map contoured at 0.03 in ChimeraX), VMAT2 with serotonin (D) (map contour 0.015), VMAT2 with histamine (E) (map contour 0.005), VMAT2 with amphetamine (F) (map contour 0.002), VMAT2 with reserpine (G) (map contour 0.03), and VMAT2 with tetrabenazine (H) (map contour 0.03). See also Figures S1-S7 and Table S1.

Monoamines function as neurotransmitters in the central nervous system, with serotonin affecting mood and emotion, histamine regulating sleep, and catecholamines (dopamine, norepinephrine, and epinephrine) influencing behavior, motor control, and cognitive processes.^1-3^ Vesicular monoamine transporter 2 (VMAT2) stores these monoamines in secretory vesicles to regulate their quantal release at synaptic clefts and prevent their uncontrolled neuronal damage (Figure 1A). Monoamines are enriched by ~10,000-fold in storage vesicles through the import by VMAT2, which is a proton antiporter driven by an outward proton gradient across the vesicular membrane.^4,5^ Monoamines also regulate blood pressure, gastric secretion, and immune responses in peripheral systems, where they are stored in large dense core vesicles of neuroendocrine and endocrine cells.^6-9^ The periphery storage of monoamines is mediated by VMAT1 and VMAT2, isoforms with high sequence similarity in the SLC18 vesicular transporter family. However, these isoforms exhibit different tissue distributions. In humans, VMAT1 is primarily located in peripheral neuroendocrine cells, whereas VMAT2 is mainly found in monoaminergic neurons and histamine-storing cells including basophils and mast cells in the immune system.^10,11^ Consistent with this distribution, the histamine affinity of VMAT2 is 30- to 140-fold greater than that of VMAT1.^10,12^ For other monoamines, VMAT2 exhibits a 2- to 8-fold higher affinity compared with VMAT1.^13,14^ However, the mechanisms governing their substrate preference and potent capability of vesicular storage remain unclear.

VMAT2 is targeted by an arsenal of therapeutic drugs and imaging reagents for the treatment and monitoring of a wide range of neurological and psychiatric disorders. Tetrabenazine, a specific VMAT2 inhibitor, has been employed in the management of hyperkinetic disorders since the 1960s, including chorea associated with Huntington’s disease and tardive dyskinesia. Radioactive derivatives of tetrabenazine, which quantify VMAT2 density in monoaminergic systems, enable the early diagnosis and monitoring of disease progression in Parkinson’s and Alzheimer’s diseases, as well as in drug addiction.^15,16^ Reserpine, which selectively inhibits both VMAT1 and VMAT2, was among the first effective drugs in treating hypertension and psychiatric disorders such as schizophrenia.^17^ Reserpine-induced monoamine depletion led to the landmark discoveries that monoamines act as neurotransmitters in the central nervous system and are associated with Parkinson’s disease.^1^ Moreover, examination of the reserpine-bound state has provided insights into the proton antiport mechanism of VMATs, as the proton gradient markedly accelerates reserpine binding^18-20^ at the cytoplasmic side,^21^ similar to the protonation-induced cytoplasmic-open transition during normal VMAT transport (Figure 1A).

Amphetamines, a group of illicit and medicinal drugs, invoke psychostimulation by inducing the release of monoamines. Amphetamine (marketed as Adderall) and methylphenidate (Ritalin) are the primary medications to treat attention deficit hyperactivity disorder, a condition impacting ~3%–5% of the population, especially juveniles.^22^ Methamphetamine is a widely abused illicit drug notorious for its addictive and neurotoxic properties,^23^ and methylenedioxymethamphetamine (ecstasy or MDMA) is a popular recreational drug. These psychostimulants function, in part, by interacting with VMAT2,^23^ as supported by the fact that amphetamines compete with reserpine binding to VMAT2^24^ and that reserpine blocks the amphetamine-induced release of monoamines.^25^ However, how amphetamines promote the vesicular release of monoamines remains unclear.^23^

Here, we report a series of cryo-electron microscopy (cryo-EM) structures of human VMAT2 in the unbound state and bound to serotonin, histamine, amphetamine, reserpine, and tetrabenazine. These structures and extensive functional studies reveal high structural flexibility of VMAT2 underlying monoamine storage and demonstrate amphetamine-induced monoamine release, providing a framework for developing targeted therapeutics for neurological disorders and substance abuse.

## RESULTS

### Structure determination

Because the full-length human VMAT2 expressed in *Pichia pastoris* showed low protein levels and inhomogeneous elution profiles during fluorescence detection size exclusion chromatography (FSEC) (Figure S1A), we removed part of the VMAT2 N-terminal region (amino acids 1–17) preceding transmembrane helix (TM) 1 and a poorly conserved, glycosylated loop (64–110) between TM1 and TM2 (named L1–2 and so forth). This deletion construct (VMAT2-del) shows a dramatic increase in protein yield and improved FSEC peak profile (Figure S1A), enabling subsequent protein purification and structure determination. Compared to the full-length VMAT2, VMAT2-del shows similar import activity for [^3^H]-serotonin and largely retains that for [^3^H]-dopamine (Figures 1B and S1B). These activities rely on a proton gradient and are compromised by bafilomycin A1, a specific inhibitor of V-type H^+^-ATPase (Figure 1B). The inhibitors of VMAT2, reserpine and tetrabenazine, also suppress the transport activities of serotonin and dopamine (Figure 1B). Similarly, the purified VMAT2-del protein reconstituted into liposomes can import FFN206, a fluorescent false neurotransmitter, and this activity is dependent on the pH gradient and is inhibitable by reserpine, indicating that VMAT2-del expressed in *Pichia* retains transport activity (Figure S1C). Taken together, the VMAT2 functionalities are preserved in the VMAT2-del construct.

Obtaining high-resolution structures of VMAT2-del was challenging because VMAT2-del is a 48 kDa monomer and lacks extramembrane domains to resolve the signal ambiguities. Although 2D classification reveals high-resolution features of VMAT2-del protein particles (Figure S2A), subsequent three-dimensional 3D reconstruction only generated a low-resolution map (Figure S2B). Interestingly, when reserpine was added before the membrane solubilization step, a portion of VMAT2-del shifted to high molecular weight (MW) on size-exclusion chromatography, suggesting the formation of a large complex (Figure S2C). SDS-PAGE of the high-MW fractions showed multiple protein bands (Figure S2D), and mass spectrometry analysis of the purified complex identified multiple components from the respiratory complex IV or cytochrome c oxidase (CcO) (Data S1). Consistent with the presence of cofactors in CcO, the high-MW fractions exhibited a yellow color (Figure S2E). Subsequent cryo-EM analyses yielded a 3.2 Å resolution map that showed clear densities of protein backbones, side chains, and distinguished CcO species containing either 11 or 9 subunits lacking COX12 and COX13^26^ (Figures 1C, S3, and S4; Table S1). In both forms, VMAT2-del is associated with the transmembrane core complex of CcO (COX1-3) (Figure S5). However, confocal microscopy imaging of HEK293 cells shows that CcO and VMAT2 are predominantly located in distinct mitochondria and vesicle-like regions,^27,28^ respectively (Figure S6A). Co-immunoprecipitation also fails to detect VMAT2-CcO interactions *in vitro* (Figure S6B). Thus, the VMAT2-CcO association is likely not physiological. Nevertheless, it provides a platform to obtain high-resolution structures of VMAT2 (Figures 1C and S4; Table S1).

In complex with CcO, VMAT2-del is captured in a lumenal-facing conformation without bound ligand (Figure 1C). Through an undetermined mechanism, reserpine prompts the complex formation but is not retained in VMAT2. Thus, we subsequently added substrates and inhibitors to the purified CcO-VMAT2-del complex prior to sample vitrification and determined the ligand-bound structures (Figures 1D-1H). Despite the CcO association, our VMAT2 structures with serotonin, tetrabenazine, and reserpine are superimposable with those reported^29-32^ (Figure S7) during the revision of our work. The new ligand-free and amphetamine-bound structures, complemented by extensive biochemical analyses in this study, provide novel insights into functional and inhibitory mechanisms of VMAT2.

### Structural change between unbound and monoamine-bound states

**Figure 2.**
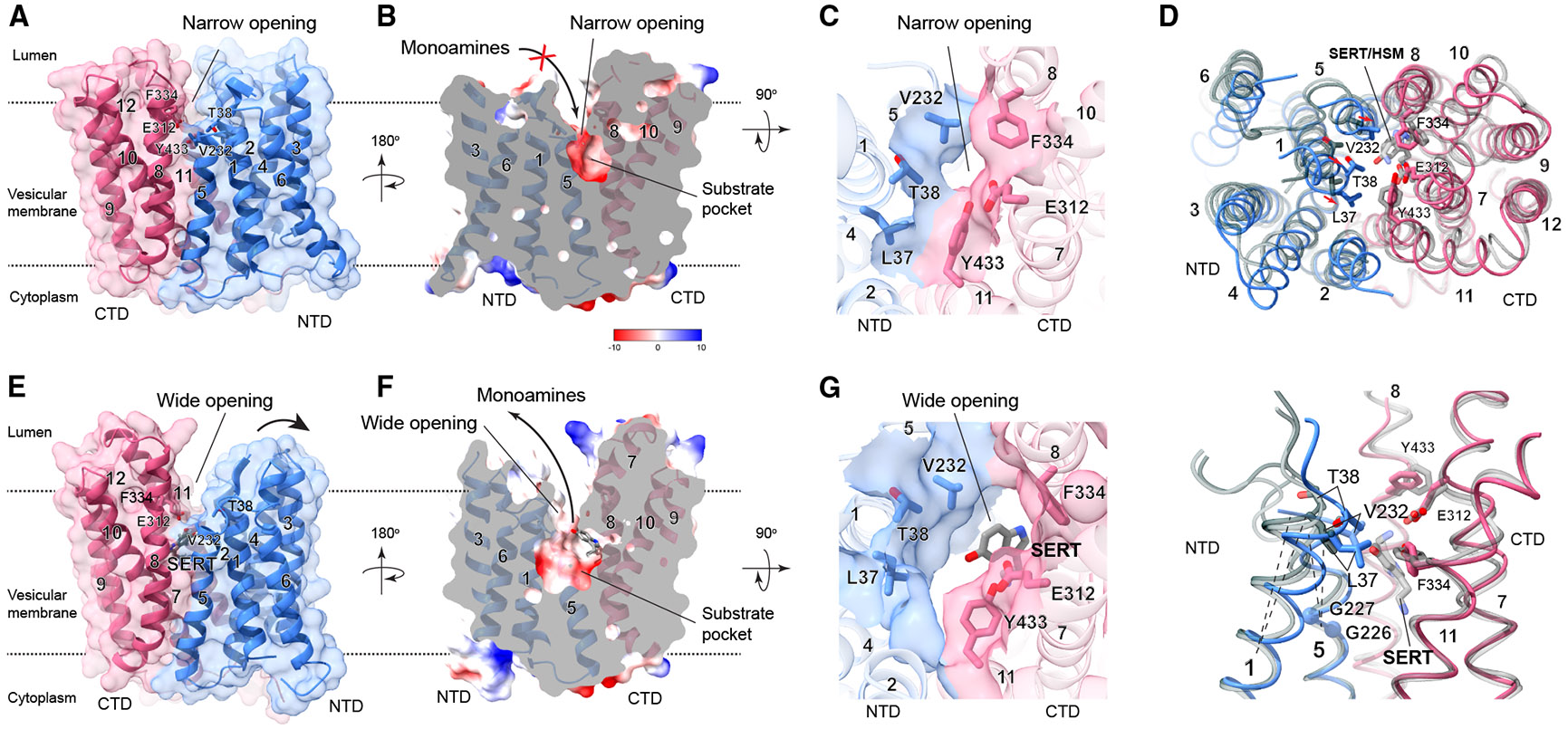
Partially occluded conformation of human VMAT2 in the unbound state and lumenal open conformation in the monoamine-bound state (A) Overall structure of the unbound state. The NTD and CTD of VMAT2 are colored in blue and red, respectively. In the absence of monoamines, the NTD and CTD primarily associate on the cytoplasmic side, resulting in a lumenal-facing conformation with a narrow opening. (B) Electrostatic surface representation showing the confined substate pocket with a narrow opening in the unbound state. (C) Top view of the narrow pocket opening. The surface and surrounding residues are colored by the NTD and CTD. (D) Structural comparison of VMAT2 in the unbound state (colored by the NTD and CTD) and with serotonin (dark gray) and histamine (light gray) bound. These structures are superimposed by the CTD, which shows fewer variations than the NTD between the structures. Top: top view of the overall structures showing relative movements of the TMs and associated residues. Bottom: side view of the key helices showing bending (dashed lines) of TM1 and TM5 in the NTD, and the movement of key residues that bind monoamines or form the narrow opening. (E–G) The enlarged substrate pocket has a wide opening in the monoamine-bound state (same representations as in (A)–(C). The wide opening facilitates the release of monoamines, and the enlarged pocket, extending below the membrane’s midpoint, permits alternative access for monoamines during the vesicular import.

VMAT2 adopts a canonical fold of the major facilitator superfamily (MFS), with twelve TMs divided into the structurally homologous N- and C-terminal domains (NTD: TM 1–6 and CTD: TM 7–12).^33,34^ In the absence of ligands, the NTD and CTD of VMAT2 primarily associate on the cytoplasmic side and slightly separate on the lumenal side (Figure 2A). Near the lumenal surface, a pocket with a narrow opening is observed (Figures 2B and 2C), indicative of a partially occluded conformation. This conformation arises from the lumenal NTD-CTD engagement between TM5 and TM8 and between TM1 and TM11, which delineates the narrow opening (Figure 2C).

VMAT2 adopts a lumenal-open conformation with either serotonin or histamine bound in the pocket, and essentially the same protein conformation is observed (Figures 2D-2F). The presence of these monoamines induces a marked broadening of the pocket’s opening (Figures 2F and 2G), in contrast to the narrow bottleneck observed in the unbound structure (Figures 2B and 2C). The bottom of this pocket also expands, forming a large chamber that extends below the midpoint of the membrane plane (Figure 2F). These enlargements arise as the lumenal side of TM1 and TM5 tilt away from the center (Figure 2D). The resulting chamber creates a substrate conduit during the rocker-switch motion, and the widened lumenal opening may facilitate the substrate release.

### Alternative recognition of serotonin and histamine

**Figure 3.**
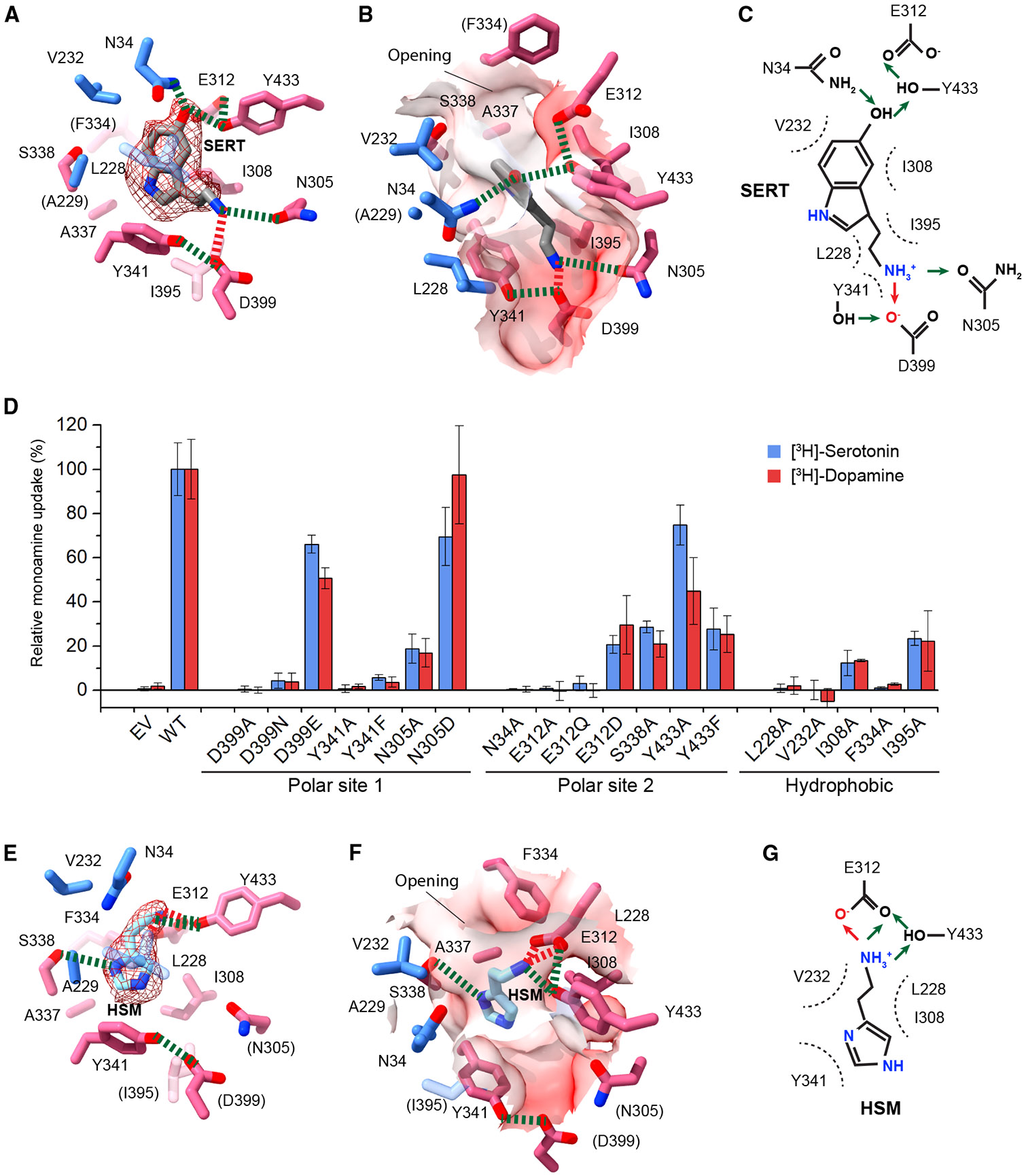
Serotonin and histamine binding in opposite orientations (A) The serotonin-binding interactions. The serotonin (SERT) molecule is shown with a density map (contoured at 0.004 in ChimeraX). The salt bridge and hydrogen bonds are shown in red and green dashed lines, respectively. Residues not directly involved in binding are indicated by parentheses. (B) Surface representation of the substrate pocket with surrounding residues. (C) Scheme of monoamine-binding residues and types of interactions, with hydrogen bond in green arrows and salt bridge in red arrows. (D) [^3^H]-serotonin and [^3^H]-dopamine uptake activities of mutants (normalized by WT) of pocket-forming residues. The similar patterns of dopamine and serotonin activities among mutants suggest similar binding interactions. The 100% uptake corresponds to 0.468 pmol/5 × 10^5^ cells for dopamine and 0.962 pmol/5 × 10^5^ cells for serotonin. Errors are SEM from three repeats. (E–G) The histamine (HSM) binding interactions. The density map in (E) is contoured at 0.004 in ChimeraX. The two negatively charged sites are defined by D399 and E312, which are hydrogen-bonded to Y341 and Y433, respectively. Either of these sites can interact with positively charged amines, thereby allowing the binding of histamine and serotonin in opposite orientations.

The serotonin binding in the substrate pocket involves a combination of polar and hydrophobic interactions (Figures 3A-3C). Serotonin possesses an indole ring with a two-carbon side chain linked to the amine group. This positively charged amine forms a salt bridge with D399 and donates a hydrogen bond to the N305 carbonyl, while the Y341 hydroxyl is hydrogen bonded with D399 carbonyls (Figure 3A). This interaction network is located at the bottom of the substrate-binding pocket (Figure 3B). Within this network, the negative charge at the D399 position is crucial, because the D399Q and D399A mutations are inactive, whereas D399E retains 70% of [^3^H]-serotonin uptake activity (Figure 3D). The Y341 hydroxyl group is also required, as the Y341F mutation results in the loss of activity. However, N305 is relatively dispensable because N305A and N305D retain 17% and 70% of the serotonin transport activities, respectively. The two-carbon sidechain and indole ring of serotonin are surrounded by several aliphatic residues, whose mutations drastically decrease the activities of [^3^H]-serotonin uptake (Figure 3D). The indole ring hydroxyl of serotonin forms hydrogen bonds with N34, E312, and Y433. Notably, these residues, together with F334, delineate the pocket opening that enlarges in the monoamine-bound state and narrows in the unbound state (Figures 2C and 2G), suggesting their key roles in the structural regulation of monoamine transport. Consistently, the N34A, F334A, and E312A mutations abolish activities of serotonin transport, although E312D and Y433 mutations are more tolerated (Figure 3D). Importantly, all mutations affecting serotonin transport show similar relative levels of activity loss for [^3^H]-dopamine transport (Figure 3D). Although we were unable to obtain the VMAT2 structure with catecholamines, these highly correlated patterns of mutant activities between dopamine and serotonin suggest that they bind in a similar manner.

Interestingly, histamine occupies the same pocket but binds in an opposite orientation, with its imidazole ring and amine group facing the pocket base and opening, respectively (Figures 3E-3G). The positively charged amine forms a salt bridge with E312 and accepts a hydrogen bond from Y433. However, histamine does not form a hydrogen bond to N305-D399-Y341 located at the pocket base. Instead, the imidazole ring is supported by a hydrophobic interaction from the pocket base. The distinct binding patterns of histamine and serotonin indicate that either of the negatively charged polar sites can bind the amine group. However, the indole ring of serotonin requires the enlarged upper portion of the pocket, whereas the relatively small imidazole ring of histamine can snugly fit into the narrower lower section, resulting in their distinct orientations (Figures 3B and 3F).

### Amphetamine binding and induced monoamine release

**Figure 4.**
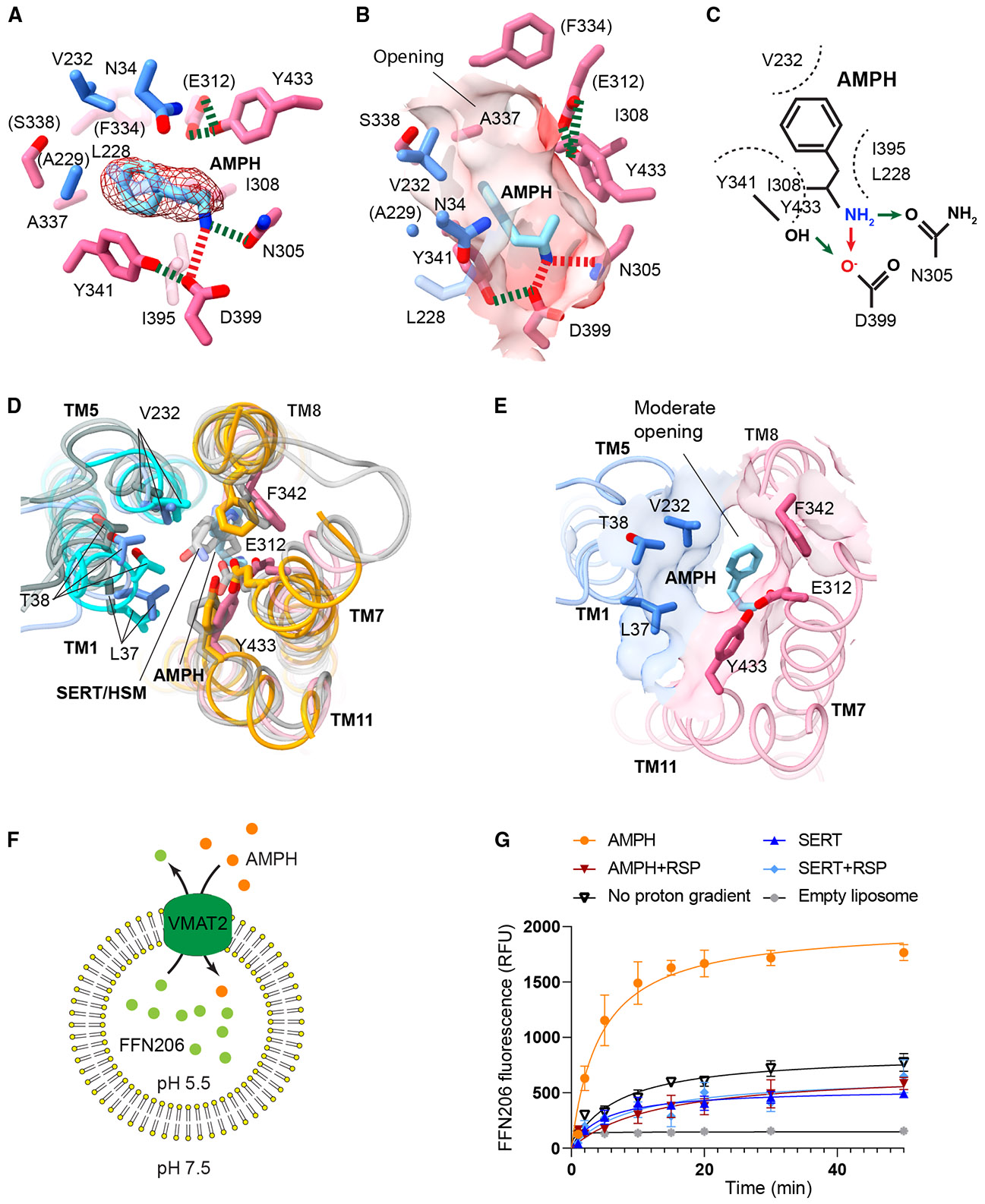
Amphetamine binding and monoamine discharge (A) The amphetamine binding interactions in the VMAT2 substate pocket. The amphetamine (AMPH) molecule is shown with the density map (contoured at 0.001 in ChimeraX), and surrounding residues are colored by the NTD and CTD. The salt bridge and hydrogen bonds are shown in red and green dashed lines, respectively. (B) Surface representation of the binding pocket with surrounding residues. (C) Scheme of amphetamine-binding residues and types of interactions, with hydrogen bond in green arrows and salt bridge in red arrows. (D) Structural comparison of VMAT2 in unbound state (blue: NTD, red: CTD) and with serotonin (dark gray), histamine (light gray), and amphetamine (cyan: NTD, orange: CTD) bound. These structures are superimposed by the CTD. Changes of key residues are indicated, which originate from the relative TM movements. (E) Surface view of the pocket opening. The size of this opening is between the monoamine-bound (Figure 2G) and -unbound states (Figure 2C). The surface and surrounding residues are colored by the NTD and CTD. (F) Scheme of the liposome assay. (G) Amphetamine promotes the release of FFN206 in liposomes. No proton gradient, pH 7.5 on both sides of proteoliposomes. Errors are SEM from three repeats. See also Figure S8.

VMAT2 binds amphetamine in the same pocket as the monoamines, albeit with differences in interactions. The chemical composition of amphetamine is α-methyl-phenethylamine, a motif shared by amphetamine-related compounds with various chemical modifications (Figure S8). The positively charged amine of amphetamine forms a salt bridge with D399 and a hydrogen bond with N305 carbonyl (Figures 4A-4C), similar to the serotonin interactions. However, amphetamine lacks a ring hydroxyl group and does not engage the E312 polar site, unlike serotonin and histamine. VMAT2 with bound amphetamine adopts a less open conformation (Figures 4D and 4E) with both TM5 and TM1 bending toward the CTD, because amphetamine lacks contact with TM1 and possesses a single ring that interacts with TM5. Consequently, the substrate binding pocket narrows and the pocket opening becomes more constricted (Figure 4E). However, the presence of the amphetamine prevents VMAT2 from reaching a partially occluded state, leaving sufficient space for amphetamine release into storage vesicles.

The traversal of amphetamine into synaptic vesicles provokes monoamine efflux, ultimately leading to psychostimulation.^23^ To investigate the underlying mechanism, we reconstituted VMAT2 into liposomes to avoid confounding factors present in cellular studies. The proteoliposomes were loaded with FFN206 to mimic neurotransmitters stored in synaptic vesicles (Figure 4F). A proton gradient across the liposome membrane was maintained by setting the internal pH to 5.5 and the external to 7.5, reflecting the pH of synaptic vesicles and cytoplasm, respectively. Upon external addition of amphetamine, we observed a substantial release of FFN206 from the liposomes (Figure 4G). As a control, liposomes lacking VMAT2 did not exhibit this release. Reserpine effectively blocked the amphetamine-induced release of FFN206, further confirming that the observed release is specifically mediated by VMAT2 activity. Interestingly, the addition of serotonin failed to induce substantial FFN206 release, confirming that this effect is specific to amphetamine. Furthermore, this release is dependent on the proton gradient, as no release is observed when the internal and external pH are equal. Taken together, these experiments demonstrate that amphetamine directly promotes neurotransmitter release, in contrast to a traditional hypothesis that the apparent release results indirectly from cytosolic monoamine accumulation due to amphetamine-induced collapse of the proton gradient.^35^

### Tetrabenazine binding

**Figure 5.**
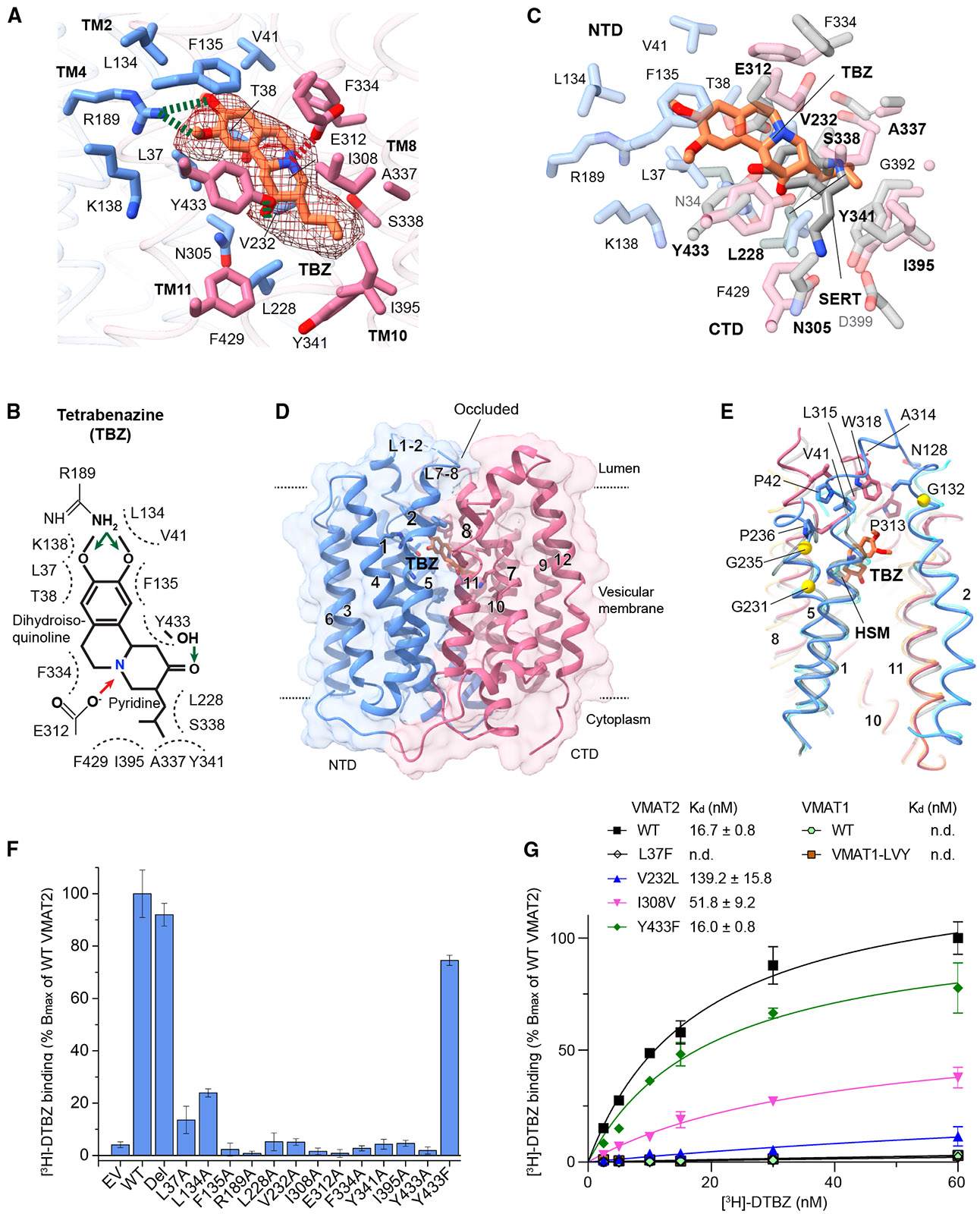
Tetrabenazine binding depends on direct interactions with VMAT2 and its structural flexibility to adopt a fully occluded conformation (A) Tetrabenazine binding interactions. The density map for tetrabenazine (TBZ) is shown (contoured at 0.03 in ChimeraX), and the surrounding residues are colored by the NTD and CTD. (B) Chemical structure of tetrabenazine and scheme of binding interactions. (C) Comparison of tetrabenazine and serotonin binding. Tetrabenazine shares interacting residues with serotonin but extends beyond the serotonin binding site, engaging with several additional residues, especially in the CTD. (D) Overall structure of VMAT2 with tetrabenazine in the lumenal-facing, fully occluded conformation. (E) Comparison of the fully occluded conformation (blue: NTD, red: CTD) with the histamine-bound, lumenal-open conformation (gray colors), and the ligand-free, partially occluded conformation (cyan: NTD, orange: CTD). The structures are superimposed by the NTD to show local differences. Residues forming the NTD-CTD interface at the lumen side of the full-occluded conformation are shown. Glycines (yellow spheres) and prolines contributing to the structural flexibility^36^ of VMAT2 are indicated. (F) [^3^H]-dihydrotetrabenazine ([^3^H]-DTBZ) binding of wild-type VMAT2 and mutants. The binding experiments are carried out at 10 nM [^3^H]-DTBZ, and the retained radioactivity is normalized to wild type. Errors are SEM from three repeats. (G) [^3^H]-DTBZ binding curves of VMAT2 and VMAT1. Distinct residues of VMAT2 and VMAT1 at the tetrabenazine binding site are replaced with the corresponding ones. VMAT1-LVY: triple substitutions of F38L, L240V, and F441Y in human VMAT1, which correspond to L37, V232, and Y433 in human VMAT2. The counts of [^3^H]-DTBZ specifically bound to each construct are normalized to the maximum number of [^3^H]-DTBZ counts (*B_max_*) bound to wild-type VMAT2. Errors are SEM from three repeats. See also Figure S9.

Tetrabenazine binds with a tilted orientation relative to the membrane plane (Figure 5A). The pyridine ring of tetrabenazine faces the membrane center, and the isobutyl sidechain reaches further than the monoamine binding site in the CTD (Figures 5B and 5C). The dihydroisoquinoline ring of tetrabenazine also interacts with more NTD residues than the monoamines. The tetrabenazine binding induces a fully occluded conformation, sealing off the lumenal opening of the substrate pocket (Figure 5D). The closure primarily arises from the interactions between the lumenal ends of TM1 and TM2 in the NTD and between those of the corresponding TM7 and TM8 in the CTD. The loops connecting these TMs, L1–2 and L7–8, also engage with each other to contribute to the closure (Figure 5E). Interactions of these flexible structural components result in a conformation more occluded than the unbound state.

Consistent with structural observations (Figure 5A), alanine mutations of nearly all tetrabenazine-interacting residues reduce [^3^H]-dihydrotetrabenazine (DTBZ, an analog of tetrabenazine) binding to the base level (Figure 5F). As exceptions, L37A and L134A retain partial DTBZ affinity, suggesting they preserve some hydrophobic interactions. Unlike the Y433A mutation, Y433F retains DTBZ binding, and consistently, Y433F alone does not confer tetrabenazine resistance in rat VMAT2.^37^ Besides Y433F, human VMAT1 and VMAT2 differ by only three additional residues, L37F, V232L, and I308V, within the tetrabenazine binding site (Figures S9 and 5A). Single substitutions of these residues in VMAT2 show that L37F nearly abolishes the DTBZ binding, while V232L and I308V reduce this binding (Figure 5G). Based on these observations, we made a combination of back substitutions in VMAT1, which, however, only slightly increases DTBZ binding affinity (Figure 5G). Thus, altering direct interacting residues alone does not endow human VMAT1 with tetrabenazine sensitivity.

### Reserpine binding and protonation-coupled alternate gating

**Figure 6.**
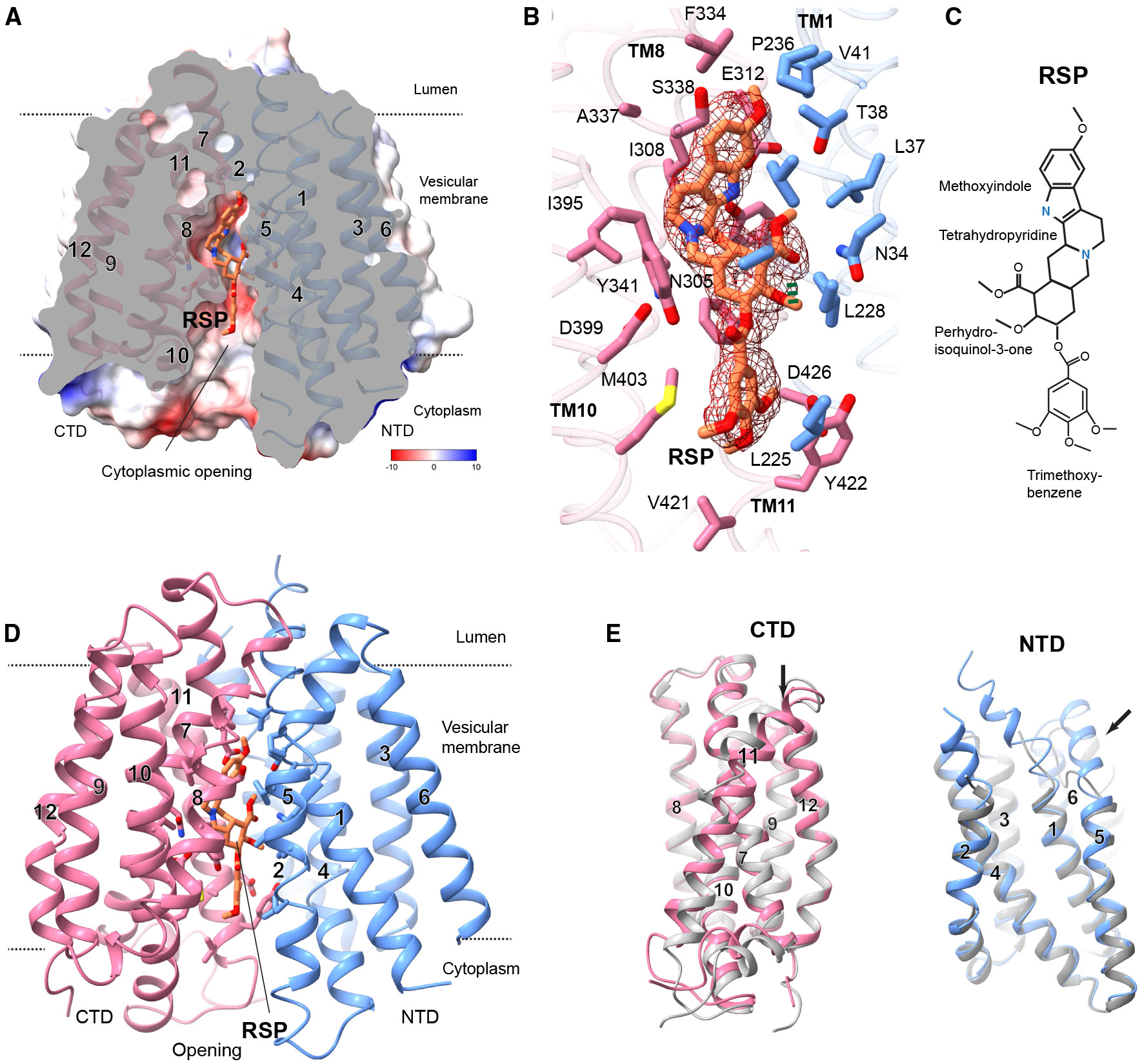
Reserpine binding in the cytoplasmic-open conformation (A) Electrostatic surface representation of the overall structure and reserpine-binding pocket in a sliced view. A large chamber is accessible from the cytoplasm and occupied by reserpine. (B) Reserpine binding interactions. The density map for reserpine (RSP) is shown (contoured at 0.03 in ChimeraX), and the surrounding residues are colored by the NTD and CTD. (C) Chemical structure of reserpine. (D) Overall structure of VMAT2 with reserpine in the cytoplasmic-open conformation. (E) Superimposition of the CTD (left) and NTD (right) domains between this cytoplasmic-open conformation and the lumenal-open conformation with bound serotonin. The close superimposition of each domain shows that the structural transition between these alternate conformations is primarily a rigid-body movement between the NTD and CTD.

**Figure 7.**
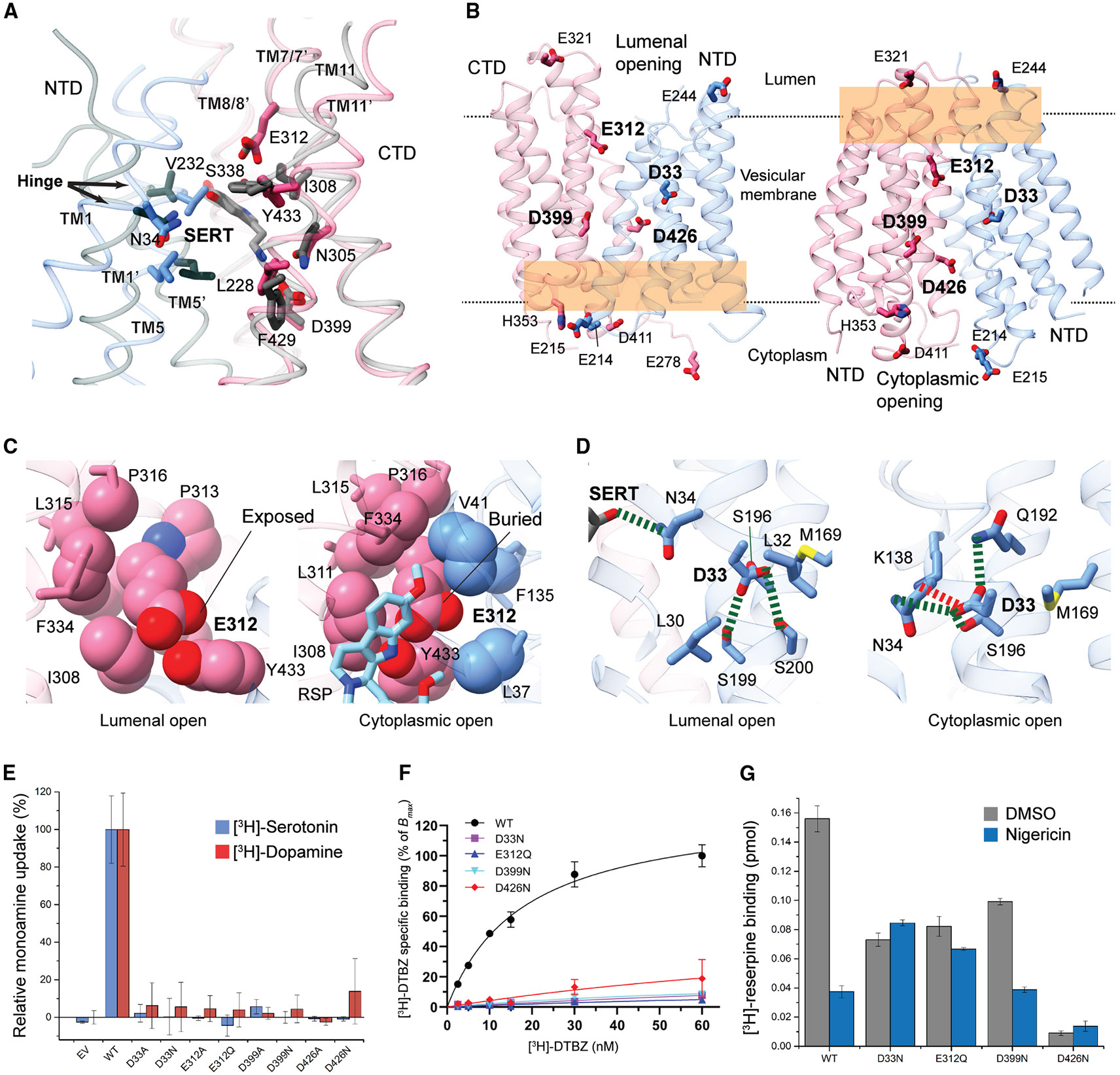
Implications of reserpine binding for the proton antiport mechanism (A) Preservation of monoamine-binding pocket in cytoplasmic-open conformation. The residues interacting with monoamines at the lumenal-open conformation (colored in gray) are largely unchanged in the cytoplasmic-open conformation (colored by NTD and CTD), which is captured by reserpine binding. (B) Mapping of protonatable residues across the alternate conformations. All acidic residues and one histidine that form the NTD-CTD gating interactions are shown. D33 is included because it has been suggested to be a protonation site during the antiport process. The orange shades indicate the alternate closure of the VMAT2 transporter that makes certain protonatable residues inaccessible from the lumenal (left) or cytoplasmic (right) opening. (C) Change of the E312 surroundings. Left: E312 is exposed to water in the lumenal-open conformation. Right: potential protonation of E312 in a hydrophobic environment. (D) Change of D33 interactions between the alternate conformations. D33 interacts with N34, a substrate-binding residue, only in the lumenal-open conformation. (E) [^3^H]-serotonin and [^3^H]-dopamine uptake activities of mutants at potential protonation sites. The 100% uptake corresponds to 0.300 pmol/5 × 10^5^ cells for dopamine and 0.659 pmol/5 × 10^5^ cells for serotonin. Errors are SEM from three repeats. (F) [^3^H]-DTBZ binding curves of wild-type VMAT2 and mutants. Errors are SEM from three repeats. (G) Reserpine binding and proton-gradient dependency (collapsed by nigericin) of neutralizing mutants at candidate protonation sites. Errors are SEM from three repeats. See also Figure S10.

VMAT2 binds reserpine in a cytoplasmic-open conformation, forming a large chamber that is mostly occupied by the reserpine molecule (Figure 6A). The binding involves extensive hydrophobic and polar interactions (Figures 6B and 6C), explaining the tight binding behavior of reserpine.^19,38^ Most reserpine-binding residues also participate in monoamine binding in the lumenal-open conformation (Figures 3 and S9), and the reserpine-binding pocket largely preserves a site suitable for monoamine binding at the import state (Figures 6A and 6D), consistent with the competitive binding of monoamine and reserpine.^39-41^ The monoamine-interacting residues in the CTD remain nearly unchanged since the rocker-switch transition occurs through rigid body movements between the NTD and CTD (Figure 6E). The NTD interacting residues, N34, L228, and V232, are also closely positioned, because the rigid-body rotation of NTD hinges around these residues (Figure 7A). Notably, reserpine recognizes the cytoplasmic-open conformation induced by protonation,^18,19^ and protonation similarly drives the cytoplasmic-open transition during the regular transport cycle.^36^ Taken together, the reserpine-bound conformation may share high structural similarity to the monoamine-import conformation, which should also maintain the same substrate pocket (Figure 7A), as expected for MFS transporters.^34,36^

Structural comparison of cytoplasmic-open and lumenal-open conformations sheds light on the mechanistic understanding of proton coupling. These alternate conformations are stabilized by “gating” interactions (Figure S10) between the NTD and CTD that regulate the transport cycle.^34^ Among the protonatable, alternate exposed residues, D33, E312, D399, and D426 (Figure 7B), the structure comparison reveals a distinct difference in E312 interactions and surroundings (Figure 7C). While Y433 donates a hydrogen bond to the carbonyl group of E312 carboxyl in both conformations, the E312 hydroxyl group is exposed to water and deprotonated only in the lumenal-open conformation. In contrast, in the cytoplasmic-open conformation, this hydroxyl group is surrounded by hydrophobic residues and may need to be protonated to mitigate the negative charge. Previous studies suggested that D33 serves as another protonation site,^42^ which forms distinct polar interactions across alternate conformations (Figure 7D). Only in the substrate-free state does it engage with the adjacent residue, N34, which is required for monoamine binding (Figure 3D). Moreover, D33 serves as the hinge point for TM1 rotation, suggesting that D33 protonation is associated with the rocker-switch transition. Additionally, the gating interactions of D399-Y341 and D426-N146 both occurred in the lumenal-open conformation and disappeared in the cytoplasmic-open conformation (Figure S10). All these acidic residues are candidates for proton translocation. Consistently, their neutralization mutations (D33N, E312Q, D399N, and D426N) or Ala mutations result in the loss of dopamine and serotonin uptake activities (Figure 7E).

To identify the protonation sites in VMAT2, we assessed whether these neutralization mutations, by mimicking the protonation state, induce the lumenal-open to cytoplasmic-open transition. All the mutations prevent the DTBZ binding (Figure 7F) at the lumenal side, suggesting their protonation destabilizes either a lumenal-facing or a tetrabenazine-binding state. Notably, D33 and D399 do not directly interact with tetrabenazine (Figure 5A). For reserpine binding at the cytoplasmic side, we found that D33N in human VMAT2 decreases the binding to ~50% of the wild type (Figure 7G), consistent with a previous study of rat VMAT2.^42^ Importantly, the reserpine binding of D33N is independent of a proton gradient, as expected for the mimic of protonation. Likewise, the E312Q binding to reserpine is independent of a proton gradient. In contrast, the reserpine binding of D399N mutant changes with the proton gradient, and D426N disrupts reserpine binding even in the presence of a proton gradient. Overall, structural and biochemical observations suggest that protonation of D33 and E312 may drive the cytoplasmic-facing transition of VMAT2.

## DISCUSSION

The cryo-EM structures of VMAT2 reveal various conformations associated with monoamine accumulation, amphetamine-induced release, and tetrabenazine and reserpine binding. The uptake of monoamines begins with their binding to VMAT2 at a cytoplasmic-open conformation induced by protonation, which may resemble the conformation observed with bound reserpine^18,19^ (Figures 6 and 7A). Monoamines should bind in a deprotonated state, as substrate binding and protonation are mutually exclusive in proton antiporters.^43^ The deprotonated VMAT2 is inclined to the lumenal-facing conformation, as supported by the cryo-EM structures obtained under neutral pH conditions that adopt this preferred orientation with or without bound substrates (Figure 2). This relatively high conformational stability may augment the substrate-binding energy utilized by MFS transporters^44^ to promote the rocker-switch transition to the lumen-facing conformation.

Following this transition, the monoamines enter a pre-release state, characterized by a large lumenal opening that permits subsequent release (Figures 2E-2G). This release is a crucial barrier for VMAT2 to overcome because the dissociation constant of monoamines needs to be > 100 mM due to their high concentration (up to 0.5 M) in storage vesicles.^45^ The lower pH (~5.5) in the vesicular lumen leads to VMAT2 protonation that displaces bound monoamines.^43^ One of the identified protonation sites, E312, also serves as the negatively charged site essential for monoamine binding (Figures 3 and 7). Therefore, E312 protonation may directly trigger monoamine release. The stoichiometry of two protons per monoamine^36,40^ may reflect the energetic requirement for accumulating monoamines at high concentrations. Thus, the second protonation site, D33, located relatively far from the monoamine-binding site (Figure 7B), is likely required to act in concert with E312 to induce the cytoplasmic-open transition. Notably, this transition constitutes the rate-limiting step of the entire transport cycle.^40^ Prior to this transition, the empty VMAT2 adopts the partially occluded conformation, in which the bottleneck of the opening measures^46^ only ~1.6 Å (Figure 2C), significantly smaller than the dimensions of monoamines. Thus, the reverse binding and transport of monoamines may be hindered. Taken together, the structural transitions promoted by protonation and deprotonation, the mutually exclusive monoamine binding, and the post-release pocket narrowing in VMAT2 together constitute a comprehensive mechanism that facilitates the uptake of monoamines and prevents their undesired export, thereby enabling their 10,000-fold accumulation in storage vesicles.

The psychostimulation of amphetamines may originate from the alteration of this protonation-coupled process by monoamine competition. Amphetamines can be imported via VMAT2, as supported by the amphetamine-bound structure (Figure 4) and the biochemical evidence that amphetamine competes with monoamine import and reserpine binding.^10,24^ Importantly, our liposome experiments demonstrate that amphetamine triggers a direct release of a fluorescent monoamine analog (Figures 4F and 4G), in contrast to the traditional weak base mechanism,^23^ suggesting that amphetamines collapse the proton gradient for uptake of monoamines and indirectly lead to their cytosolic accumulation. Thus, a new mechanism can be postulated. Unlike monoamines, amphetamine binding does not involve E312, a key protonation site (Figure 7). As a result, protonation-induced amphetamine release is likely less effective. Instead, amphetamine may be released through competitive binding of monoamines that are present at an extremely high concentration (up to 0.5 M) in storage vesicles (Figure 4F); this competitive replacement is consistent with the fact that amphetamine displaces monoamines in a stoichiometric manner.^47,48^ Subsequently, VMAT2 with bound monoamine may revert to the cytoplasmic-open conformation. The monoamine is then released into the cytoplasm and further dissipated into the synaptic space to cause psychostimulation. This proposed mechanism may be shared by psychostimulants that have minor to moderate alterations from amphetamine (Figure S8), such as methamphetamine and MDMA, showing VMAT2 affinities comparable to that of amphetamine.^10,24^ VMAT2 inhibitors blocking this mechanism, such as lobeline, may decrease monoamine release,^49,50^ offering the potential to develop therapeutics to treat amphetamine-related drug abuse.

A major difference between the two VMAT isoforms is their differing affinities for tetrabenazine and histamine. Structurally, human VMAT2 exhibits relatively high flexibility, as evidenced by the spectrum of conformations readily biased by ligand binding. In contrast, such ligand-induced flexibility was not observed in our recent human VMAT1 structures.^51^ Genetic screens or targeted mutagenesis examining tetrabenazine resistance identified residues modulating TM flexibility or inter-TM interactions,^10,12,37,52-54^ potentially enabling VMAT2 to adopt the fully occluded conformation required for tetrabenazine binding. Many of these resistance mutations mapped to proline, glycine, or adjacent residues near the lumenal side of TMs^52^ (Figure 5E), underscoring the importance of local flexibility. Additionally, P236, corresponding to A244 in VMAT1, is key to the histamine affinity of VMAT2^54^ (Figure 5E). The higher structural flexibility of VMAT2 may enhance substrate adaptability, potentially explaining its higher affinities for other monoamines compared to VMAT1.^13,14^

Owing to their pivotal roles in neurotransmission and endocrine function, VMATs are among the primary pharmacological targets for neurological and psychiatric disorder management. Amid a recent surge of groundbreaking cryo-EM structures of VMATs with substrates and inhibitors,^29-32,51^ here, we show that VMAT2 in the lumenal-facing conformation is readily biased by the binding of different substrates and tetrabenazine. This structural flexibility, not observed for VMAT1,^51^ may underlie the high tetrabenazine and histamine affinities of VMAT2. Furthermore, we demonstrate that amphetamine directly induces false transmitter release via VMAT2 in a minimal liposomal system, and structural observations suggest a potential interplay between protonation and amphetamine-induced monoamine release. These insights lay the foundation for developing innovative treatments for neurodegenerative disorders and psychostimulant abuse.

### Limitations of the study

The structural flexibility of VMAT2 appears to underlie its high tetrabenazine sensitivity and ability to transport histamine, although the specific regions conferring this flexibility were not defined here. Possible targets include loop regions or flexible points in TMs that bend in different ligand-bound structures. If a limited number of such substitutions in VMAT1 confer tetrabenazine sensitivity and enable histamine transport, this would support the hypothesis that structural flexibility underlies these VMAT2-specific functions.

We propose an amphetamine-induced release mechanism that bypasses protonation coupling. Unlike monoamines, amphetamines lack interaction with E312, a key protonation site that may trigger monoamine release into the vesicular lumen. Tests using amphetamine analogs that differ in their ability to engage E312 are needed to assess whether this site is essential to the release mechanism underlying psychostimulation.

## STAR★METHODS

### EXPERIMENTAL MODEL AND STUDY PARTICIPANT DETAILS

#### Cell lines and microbe strains

HEK 293T cells (ATCC) were maintained in Dulbecco’s minimal essential medium (DMEM) supplemented with 10% (v/v) fetal bovine serum (FBS) and 100 U/mL penicillin-streptomycin at 37°C with 5% CO_2_ in a humidified incubator. *Pichia pastoris* cells (ATCC) used for protein expression and purification were cultured in the minimal glycerol media (1.2% glycerol, 0.34% yeast nitrogen base, 1% ammonium sulfate, 0.4 mg/mL biotin, and 100 mM potassium phosphate, pH 6.0) at 30°C for 24 h, then transferred to the minimal methanol media (0.34% yeast nitrogen base, 1% ammonium sulfate, 0.4 mg/mL biotin, and 200 mM potassium phosphate, pH 6.0) at 30°C for 48 h. *Escherichia coli* DH5α (NEB) used for cloning were cultured in LB media with appropriate antibiotics at 37°C. These cell lines and microbe strains were authenticated by commercial vendors.

### METHOD DETAILS

#### Constructs

Human VMAT2 (UniProt: Q05940; *SLC18A2* gene) in a pDONR-221 plasmid was acquired from DNASU and used as the template for subcloning. For expression in *Pichia pastoris*, VMAT2 was cloned into a modified pPICZ-B vector (Thermo Fisher Scientific) under a methanol-inducible promoter. The expression construct contains a C-terminal PreScission protease cleavage site, followed by green fluorescent protein (GFP) and a FLAG tag. For expression in HEK293T cells, VMAT2 was cloned into a modified pBudCE4.1 vector also containing the C-terminal PreScission site, GFP, and FLAG tag. To enhance protein stability, VMAT2 was systematically truncated at its N-terminal, C-terminal, and loops between TMs. These deletion constructs, as well as VMAT2 mutants and constructs for the uptake assays, [^3^H]-DTBZ binding assay, coimmunoprecipitation, immunofluorescence imaging and mass spectrometry (MS) analysis were generated using standard PCR-based methods. All nucleotide sequences were verified by DNA sequencing.

#### Protein expression and FSEC analysis

The pPICZ-B plasmids encoding VMAT2 constructs were linearized by Pme I and transformed into *Pichia pastoris* by electroporation. Transformants were selected by Zeocin resistance on yeast extract peptone dextrose medium with sorbitol (YPDS) agar plates. The expression levels and elution profiles of resistant clones were compared through fluorescence-detection size-exclusion chromatography (FSEC) (λex/λem: 515 nm/530 nm) analysis. Briefly, the *Pichia* cells were disrupted using a mixer mill and the cell membrane was dissolved in 2% n-dodecyl-β-D-maltoside (DDM, Anatrace). After removing the cell debris, the supernatant was loaded onto a Superose 6 size-exclusion column, and the elution profile was monitored by GFP fluorescence. Constructs with monodispersed elution profiles and high expression levels were selected and stored at −80°C.

For large-scale protein expression, *Pichia* cells from the FSEC selection were grown in the buffered minimal glycerol media (1.2% glycerol, 0.34% yeast nitrogen base, 1% ammonium sulfate, 0.4 mg/mL biotin, and 100 mM potassium phosphate, pH 6.0) at 30°C for 24 h. Subsequently, the growth media was replaced with buffered minimal methanol media (0.34% yeast nitrogen base, 1% ammonium sulfate, 0.4 mg/mL biotin, and 200 mM potassium phosphate, pH 6.0), and protein expression was induced with 0.7% methanol. After 2 days at 25°C, the cells were harvested by centrifugation and flash frozen in liquid nitrogen.

#### Protein purification

Frozen *Pichia* cells (30 g) were disrupted by milling (Retsch PM100) and resuspended in a lysis buffer containing 150 mM NaCl, 0.1 mM reserpine (Sigma), and 20 mM Tris, pH 8.0, supplemented with protease inhibitor cocktail (Sigma, 1:1000). The cell membranes were subsequently lysed in a sonicator at 50% power for 35 min with 1 s/2 s on/off cycles in a water-ice bath. After the lysis, cell debris were pelleted by centrifugation at 4000*g* for 15 min. The supernatant was subjected to centrifugation at 4°C, 195,000 g for 30 min. The pelleted membranes were vortexed into 50 mL of resuspension buffer (150 mM NaCl, 0.4 mM reserpine, and 20 mM Tris, pH 8.0), followed by Dounce homogenization on ice. Subsequently, 1.0 g n-dodecyl-b-D-maltoside (DDM, Anatrace; 2% (w/v) final concentration) was added to solubilize the membranes by stirring for 2 h at 4°C. After centrifugation at 195,000 g for 30 min, the supernatant was incubated with 3 mL of anti-FLAG M2 resins (Sigma) for 1 h at 4°C. The resins were washed with 50 mL wash buffer containing 150 mM NaCl, 0.08% (w/v) lauryl maltose neopentyl glycol (LMNG, Anatrace), and 20 mM Tris-HCl, pH 8.0. The protein was eluted with 15 mL of elution buffer containing 150 mM NaCl, 0.02% (w/v) LMNG, and 20 mM Tris-HCl, pH 8.0, supplemented with 200 μg/mL PreScission protease. The eluent was concentrated using an Amicon Ultra-15 centrifugal filter unit (MW cutoff 100 kDa) and loaded into a Superose 6 Increase 10/300 gel filtration column (GE Healthcare) in 150 mM NaCl, 0.01% (w/v) LMNG, 0.0033% (w/v) glyco-diosgenin (GDN, Anatrace), and 20 mM Tris-HCl, pH 8.0. The peak fractions were collected and concentrated for cryo-EM and MS analyses.

#### Peptide mapping

The purified protein was lyophilized and 75 μg of the protein was resuspended in 130 μL denaturation buffer containing 8 M Urea, 50 mM Tris-HCl, pH 7.5. To reduce disulfide bonds, 10 μL of 50 mM dithiothreitol (DTT) was added to the sample, followed by incubation at 55°C for 30 min. Subsequently alkylation was carried out by the addition of 15 μL 100 mM iodoacetamide and incubation in darkness at room temperature (RT) for 30 min. The reaction was then quenched by the addition of 5 μL of 50 mM DTT with 5 min incubation at RT. Sample volume was adjusted to 1 mL with 50 mM Tris-HCl, pH 7.5. Proteolytic digestion was initiated by the addition of 5 μL trypsin and/or chymotrypsin (1 mg/mL in 50 mM Tris-HCl, pH 7.5, 10 mM CaCl_2_). The digestion was carried out at 37°C overnight and quenched with 4 μL formic acid (FA). Samples were desalted using Sep-Pak tC18 1cc Vac Cartridges, 50 mg Sorbent per cartridge, 37–55 μm (Waters), and then lyophilized in a Savant SpeedVac Concentrator (Thermo Fisher Scientific). Samples were then reconstituted in 20 μL 2% ACN, 0.1% FA in water. The peptide concentration was measured with a NanoDrop Microvolume UV-Vis Spectrophotometer (Thermo Fisher Scientific), and then diluted in the same buffer to a concentration of 0.05 mg/mL.

The digested peptides were analyzed by online nanoflow liquid chromatography-tandem mass spectrometry (LC-MS/MS) using an Orbitrap Q Exactive Plus (Thermo Fisher Scientific) with a Nanospray Flex source (Thermo Fisher Scientific) coupled online to an UltiMate 3000 RSLCnano System (Thermo Fisher Scientific). The sample (5 μL) was loaded onto on a microcapillary column containing an integrated electrospray emitter tip, packed with ReproSil-Pur C18-AQ 3 μm beads (Dr. Maisch GmbH, ESI Source Solutions) at RT. The HPLC solvent A was 0.1% FA in H_2_O, and solvent B was 80% ACN, 20% H_2_O, 0.1% FA. Samples were measured using a 165 min MS method, at a flow rate of 0.250 μL/min with the following gradient profile: (min:%B) 0:2; 10:2; 15:15; 60:42; 65:60; 92:95; 97:95; 107:95; 110:2; 120:2; 121:95; 126:95; 127:2; 132:2; 133:95; 138:95; 139:2; 144:2; 145:95; 154:95; 155:2; 165:2. The Orbitrap Q Exactive Plus was operated in data-dependent mode acquiring HCD MS/MS scans (resolution = 35,000) after each MS1 scan (resolution = 70,000) on the most abundant ions using an MS1 target of 3 × 10^6^ and an MS2 target of 1 × 10^5^. The maximum ion time utilized for MS/MS scans was 120 ms and the HCD normalized collision energy was set to 32. The dynamic exclusion time was set to 40 s, with the peptide match function disabled and the isotope exclusion function enabled. Identification of proteins contained within samples was conducted using PMI Byonic (Protein Metrics), with parent and fragment mass tolerance of 20 ppm, fully specific trypsin digestion, and 8 kDa maximum precursor mass. Carbamidomethylation at cysteine was the only fixed modification, and variable modifications were protein N-terminal pyro-Glu formation (E and D), deamidation (N), and oxidization (M, W).

#### Cell culture and transfection

HEK293T cells were cultured in Dulbecco’s Modified Eagle Media with 10% fetal bovine serum and penicillin–streptomycin (100 U/mL) in a CO_2_ incubator at 37°C. For [^3^H]-dopamine and [^3^H]-serotonin uptake assay, [^3^H]-dihydrotetrabenazine ([^3^H]-DTBZ) binding assay, turbo-ID, and Co-IP assay, the cells were grown in 6-well plates to 70–80% confluence and transfected with 1.5 μg of plasmid DNA coding for VMAT2 constructs using the polyethylenimine (PEI) transfection reagent (Polysciences, Inc). For immunostaining assay, cells were plated on poly-L-lysine-coated glass coverslips at a density of at 1 × 10^5^ cells/mL, then cells were grown in 6-well plates to 40–50% confluence and then transfected with 2 μg plasmid.

#### Vesicular uptake of [^3^H]-dopamine and [^3^H]-serotonin

The monoamine uptake and storage assays were adapted from a previous report^61^ with the following modifications. Briefly, transfected HEK293T cells were either untreated or incubated in the medium containing 4 μM reserpine, tetrabenazine, or bafilomycin A for 2 h. The cells were then trypsin-detached and an aliquot was taken for the FSEC estimation of protein expression level. The remaining cells were aliquoted to MultiScreenHTS 96-Well Filter Plates (Sigma) at 5 × 10^5^ cells/well, and washed once with an uptake buffer containing 110 mM Na-K-tartrate, 5 mM glucose, 0.2% (wt/vol) bovine serum albumin (BSA), 5 mM MgCl_2_, and 20 mM K-HEPES, pH 7.4. Cells were then permeabilized with 10 μM digitonin in the uptake buffer for 10 min at 37°C. This buffer was then replaced by the uptake buffer supplemented with 5 mM ATP, 1 mM ascorbic acid, and ~20 nM [^3^H]-serotonin (40.7 Ci/mmol, PerkinElmer) or [^3^H]-dopamine (45.6 Ci/mmol, PerkinElmer). Nonspecific accumulation of [^3^H]-serotonin or [^3^H]-dopamine was measured in the presence of 4 μM reserpine. At the end of incubation (10 min for [^3^H]-serotonin and 25 min for [^3^H]-dopamine), the reaction buffer was removed and the cells were washed twice with ice-cold uptake buffer containing 2 mM MgSO_4_ (without MgCl_2_). The filter paper containing cells from each well was transferred to a microbeta vial and dissolved in a biodegradable scintillation cocktail (Research Products International). Radioactivity was quantified by a liquid scintillation counter (Hitachi Aloka LSC8000). The [^3^H]-se-rotonin and [^3^H]-dopamine uptake activities of wildtype VMAT2 or mutants were normalized by protein expression levels (determined by FSEC).

#### Liposomal uptake of FFN206

To prepare proteoliposomes, VMAT2 protein was purified in buffers containing 0.01% DDM and 150 mM NaCl in either 20 mM Tris-HCl, pH 7.5 or 20 mM Bis-Tris, pH 5.5, and concentrated to ~10 mg/mL. Polar brain lipids were resuspended at 10 mg/mL in 500 μL of reconstitution buffers containing 1.2% octyl glucoside and 150 mM NaCl in the respective pH buffers. The lipid suspension was sonicated and mixed with purified VMAT2 at a protein-to-lipid ratio of 1:40 (w/w) under matched pH conditions. Detergents were removed by overnight incubation with Bio-Beads SM2 (400 mg/mL). Proteoliposomes were collected by ultracentrifugation at 100,000 × g for 1 h and resuspended in the respective reconstitution buffers.

FFN206 import assays were conducted by adding 2 μL of proteoliposomes to 98 μL of reaction buffer containing 150 mM NaCl and 20 mM Tris-HCl, pH 7.5, supplemented with varying concentrations of FFN206, with or without 4 μM reserpine. After a 10 min incubation, the proteoliposomes were passed through a G-50 desalting column to remove unimported FFN206. Proteoliposomes were then collected by ultracentrifugation at 100,000 × g for 1 h and solubilized in reaction buffer containing 10% Triton X-100. FFN206 fluorescence was measured using a SpectraMax M5 plate reader at 360 nm excitation and 460 nm emission.

#### Amphetamine induced FFN206 release in liposomes

FFN206 was loaded into the proteoliposomes by mixing 1:1 (v/v) with 2 mM FFN206 in a buffer containing 150 mM NaCl and either 20 mM Tris-HCl, pH 7.5 or 20 mM Bis-Tris, pH 5.5, followed by three freeze–thaw cycles and sonication. The proteoliposomes were passed through a G-50 desalting column to remove unincorporated FFN206, collected by ultracentrifugation at 100,000 × g for 1 h, and resuspended in the respective buffers. For the FFN206 release assay, 20 μL of proteoliposomes were diluted into 200 μL of assay buffer containing 150 mM NaCl, 3 mM KCl, 10 mM MgCl_2_, 10 mM glucose, 10 mM EGTA, 200 μM ascorbic acid, and 20 mM Tris-HCl, pH 7.5. Amphetamine or serotonin was added to the assay buffer to a final concentration of 10 μM, and samples were incubated at room temperature for varying durations. To inhibit VMAT2 activity, 4 μM reserpine was pre-incubated in the assay buffer for 90 min prior to amphetamine addition. The assay mixture was filtered through 0.22 μm GSWP filters (Millipore), and the FFN206 fluorescence of the flow-through was measured.

#### [^3^H]-DTBZ binding assay

The assay condition was adapted from the [^3^H]-DTBZ binding assay previously reported^42^ with the following modifications. Briefly, HEK293 cells were trypsinized and aliquoted to MultiScreenHTS 96-Well Filter Plates (Sigma) after 24 h of transfection. Cells were washed once with a wash buffer containing 110 mM Na-K-tartrate, 5 mM glucose, 0.2% (wt/vol) BSA, 5 mM MgCl_2_, and 20 mM K-HEPES, pH 7.4. Cells were subsequently permeabilized in the wash buffer supplemented with 10 μM digitonin for 10 min at 37°C. The buffer was then removed, and a binding buffer containing 5 mM ATP, 1 mM ascorbic acid, 10 nM or varying concentrations of [^3^H]-DTBZ (20 Ci/mmol, American Radiolabeled Chemicals, Inc.) was added to initiate the binding reaction. Nonspecific accumulation of [^3^H]-DTBZ was measured in the presence of 4 μM reserpine. The reaction was terminated by aspirating the reaction buffer after 20 min incubation, and the cells were washed twice with ice-cold wash buffer containing 2 mM MgSO_4_ (without MgCl_2_). The cells were spotted on filter paper, collected, and dissolved in 2 mL of biodegradable scintillation cocktail (Research Products International) in a beta-counter compatible vial, and radioactivity was counted with a liquid scintillation counter (Hatachi Aloka LSC8000) after incubation for 2 h. The counts of [^3^H]-DTBZ bound to wildtype VMAT2 or mutants was first normalized to protein expressing levels determined by FSEC, and then normalized to the maximum number of [^3^H]-DTBZ counts (*B_max_*) bound to wildtype VMAT2. The *K*_d_ value was fitted with classical one-site specific binding model using GraphPad Prism.

#### [^3^H]-reserpine binding assay

The [^3^H]-reserpine binding assay were adapted from a previous report^42^ with the following modifications. Briefly, HEK293T cells in 15 cm-well plates were transfected with 20 μg plasmids encoding the VMAT1 constructs. After 24 h of transfection, HEK293T cells were treated 10 μM nigericin that collapses the proton gradient or with the same volume of DMSO for 2 h. Subsequently, the cells were trypsin-detached and an aliquot was taken for the FSEC estimation of the protein expression level. The remaining cells were resuspended with 5 mL wash buffer (140 mM KCl, 5 mM glucose, 5 mM MgCl2, 20 mM HEPES, pH 7.4) supplemented with 5 mM ATP and permeabilized by the addition of 100 μM digitonin for 5 min. The binding reaction was initiated by the addition of 5 nM [^3^H]-reserpine (ViTrax Radiochemicals; 20 Ci/mmol), and cells were then incubated for 15 min at 37°C. Reactions were stopped by quickly diluting the cells with ice-cold binding buffer and collected by brief centrifugation (12,700 × g, 1 min). The cells were then solubilized in the wash buffer containing 2% (wt/vol) DDM, 15 μg/mL DNaseI (Sigma), and protease inhibitor mixture (Sigma). After 1 h of shaking at 4°C, cells were centrifuged for 10 min at 18,900 × g and incubated with Ni-Magbeads (GeneScript) for 1 h at 4°C. The beads were washed twice with wash buffer containing 0.08% DDM, and incubated with 650 μL of the same buffer with 450 mM imidazole at room temperature for 20 min. After the elution, 200 μL aliquots were measured for radioactivity using liquid scintillation.

#### Immunoprecipitation

After 24 h transfection, HEK293T cells were collected and resuspended in cold RIPA buffer (50 mM Tris pH 7.5, 150 mM NaCl, 1% Triton X-100, 1 mM PMSF, and 0.1% protease inhibitor cocktail). Extracts were immunoprecipitated with anti-FLAG M2 resin (Sigma) at 4°C for 2 h. After wash with the RIPA buffer for three times, the captured proteins were eluted by 20 μL SDS–PAGE loading dye and detected by immunoblotting on polyvinylidene fluoride membranes. The western blot used rabbit monoclonal antibody against COX4 (ZRB1593; Sigma, 1:1000) or rabbit monoclonal antibody against Flag (D6W5B, Cell Signaling Technology, 1:1000) as the primary antibody, and HPR-conjugated goat anti-rabbit antibody as the secondary antibody, followed by exposure to autoradiography film.

#### Immunofluorescence imaging

After 48 h of transfection, cells were permeabilized in phosphate-buffered saline (PBS) with 0.2% Triton X-100 at 4°C for 20 min and blocked with 10% donkey serum at room temperature for 1h, followed by incubation with rabbit anti-COX4 antibody (ZRB1593; Sigma, 1:500) at 4°C for 12 h. The secondary antibody used goat-*anti*-rabbit conjugated to Alexa Fluor 568 (SAB4600310; Sigma) at 1:1000 dilution. The coverslips were washed with PBS and mounted with antifade mounting medium (VectorLabs) onto glass slides. Fluorescence images were acquired using a Zeiss LSM 880 laser-scanning confocal microscope. The cells were captured using 40 ×1.4 oil objective with pinhole setting to 1 Airy unit.

#### Cryo-EM sample preparation and data acquisition

For unbound/monoamine and amphetamine-bound samples, the purified, reserpine stabilized CcO-VMAT2-del complex at ~7.5 mg/mL were incubated with 3 mM serotonin (Sigma), 10 mM histamine (Sigma), or 1 mM amphetamine (Sigma) for 1 h on ice. For reserpine and TBZ-bound samples, the purified CcO-VMAT2-del complex were incubated with 100 μM reserpine (Sigma) or 100 μM TBZ (MCE) for 2 h on ice and further purified by size exclusion chromatography on a Superdex 200 Increase 10/300 column (GE Healthcare) in 150 mM NaCl, 0.01% (w/v) LMNG, 0.0033% (w/v) GDN, and 20 mM Tris-HCl, pH 8.0. The peak fractions were collected and concentrated to ~8 mg/mL for the preparation of cryo-EM grids. Subsequently, 3 μL of the samples were added to glow-discharged holey carbon, 300 mesh R1.2/1.3 Au grids (Quantifoil). The grids were incubated for 20 s and blotted for 3 s at 8°C and 90% humidity, and then plunge-frozen into liquid ethane using an EM GP2 automatic plunge freezer (Leica).

Cryo-grids were screened on Glacios (FEI) electron microscope operating at 200 kV equipped with a Falcon IV detector (Thermo Fisher Scientific), and grids in good quality were transferred into Titan Krios (FEI) operated at 300 kV for data acquisition. Images were collected using the K3 Summit detector (Gatan) in CDS mode with a BioQuantum GIF energy filter (slit width of 20 eV) at the Hormel Institute, University of Minnesota. The data collection was performed using the EPU software (Thermo Fisher Scientific) with a pixel size of 1.1 Å (magnification of 81,000×) or 0.664 Å (magnification of 130,000×) and a nominal defocus value between −1.0 to −2.0 μm. Each image consists of 40 dose-framed fractions and was recorded with a dose rate of 25 e^−^/Å ^2^/s and a total dose of 50 e^−^/Å^2^. Cryo-EM data collection statistics are summarized in Extended Data S1.

#### Cryo-EM data processing

A total of 4614 movies were collected for CcO-VMAT2-del complex (incubated with reserpine only) and subjected to patch-based motion correction and CTF estimation in cryoSPARC v4.^55^ Images with the defocus values outside of −0.6 to −2.8 μm or CTF fit resolutions worse than 5 Å were excluded from further steps. Particles were then automatically picked from a small set of movies using Blob picker with 320 pixels of box size. Two rounds of 2D classification were performed to remove ice, contaminants and aggregates, yielding 265,130 particles. These particles were subjected to 2D classification, and the classes from 102,619 particles corresponding to intact CcO-VMAT2-del complex were used as templates for further template-based particle picking. The selected particles were subjected to 2D classification, resulting in 444,171 particles corresponding to the CcO-VMAT2-del complex. These particles were used to generate an *ab initio* model of five maps with C1 symmetry. The initial models were set as the starting references for heterogeneous refinement. The selected 3D classes were then subjected to further homogeneous, non-uniform and CTF refinements, generating two different maps at 3.20 Å and 3.26 Å resolution, respectively, as determined by the gold-standard measure of Fourier shell correlation using a cutoff of 0.143. Serotonin-bound (4,437 movies; 117,692 particles), histamine-bound (4,007 movies; 84,534 particles), amphetamine-bound (4,102 movies; 114,678 particles), TBZ-bound (3506 movies; 116,216 particles), and reserpine-bound (6520 movies; 61,624 particles) datasets were processed using a similar procedure and yielded reconstructions at 3.28 Å, 3.31 Å, 3.25 Å, 3.18 Å and 3.14 Å, respectively, as estimated by gold-standard Fourier shell correlation. Local resolution variations were estimated from the two half-maps in cryoSPARC. The maps were sharpened using DeepEMhancer.^58^

#### Model building and refinement

The VMAT2 structure predicted by AlphaFold (ID: AF-Q05940-F1)^62^ and the structure of cytochrome *c* oxidase from *Saccharomyces cerevisiae* (6ymy)^63^ were used as the initial model for the ligand-free CcO-VMAT2-del complex and docked into the cryo-EM map using UCSF Chimera. The resulting CcO-VMAT2-del complex model was manually rebuilt in COOT^56^ and further refined by real space refinement in PHENIX.^57^ This refined model was used as the initial model for CcO-VMAT2-del complex structures with bound substrates and inhibitors, which were fitted into the density maps using COOT. The resulting models were manually rebuilt in COOT and further refined by real space refinement in PHENIX. The model stereochemistry was evaluated using the comprehensive validation (cryo-EM) utility in PHENIX. Structural figures were generated using ChimeraX.^60^

### QUANTIFICATION AND STATISTICAL ANALYSIS

All statistical details of experiments can be found in figure legends.

## Supplementary Material

1

2

Supplemental information can be found online at https://doi.org/10.1016/j.celrep.2025.116490.

## Figures and Tables

**Table:** KEY RESOURCES TABLE

REAGENT or RESOURCE	SOURCE	IDENTIFIER
Antibodies		
Anti FLAG tag rabbit monoclonal antibody	Cell Signaling Technology	Cat# 14793S; RRID: AB_2572291
Anti COX4 rabbit monoclonal antibody	Sigma	Cat# ZRB1593
HPR-conjugated goat anti-rabbit antibody	Cell Signaling Technology	Cat# 7074S; RRID: AB_2099233
CF568-conjugated goat anti-rabbit antibody	Sigma	Cat# SAB4600310; RRID: AB_2833002
Bacterial and virus strains		
*E. coli* DH5α	New England Biolabs	Cat# C2987H
Chemicals, peptides, and recombinant proteins		
Dulbecco’s Modified Eagle Media	GIBCO	Cat# 11965084
Fetal bovine serum (FBS)	GIBCO	Cat# 10091148
Penicillin-Streptomycin (10,000 U/mL)	GIBCO	Cat# 15140163
LB medium	BD	Cat# 214906
Polyethylenimine (PEI)	Polysciences	Cat# 02371
Trypsin–EDTA, 0.05%	Thermo Fisher Sciencetific	Cat# 25300054
Zeocin	Thermo Fisher Sciencetific	Cat# R25001
ECL western blotting substrate	Thermo Fisher Sciencetific	Cat# 32109
Biotin	Thermo Fisher Sciencetific	Cat# 230090010
n-Dodecyl-b-D-maltopyranoside (DDM)	Anatrace	Cat# D310
glyco-diosgenin (GDN)	Anatrace	Cat# GDN101
Lauryl maltose neopentyl glycol (LMNG)	Anatrace	Cat# NG310
[^3^H]-dihydrotetrabenazine	American Radiolabeled Chemicals	Cat# ART0496
[^3^H]-serotonin	Perkin-Elmer	Cat# NET498001MC
[^3^H]-dopamine	Perkin-Elmer	Cat# NET673001MC
Tetrabenazine	MCE	Cat# HY-B0590B
Reserpine	Sigma	Cat# 83580-1G
Serotonin	Sigma	Cat# H9523-250MG
Histamine	Sigma	Cat# H7125-1G
Amphetamine	Sigma	Cat# A-007-1ML
Ampicillin	Sigma	Cat# A9518-25G
Sodium chloride	Sigma	Cat# S3014
Trizma base	Sigma	Cat# T6606-5MG
Yeast nitrogen base	Sigma	Cat# Y1251-1KG
Glycerol	Sigma	Cat# G7893-500ML
Ammonium sulfate	Sigma	Cat# A5132-5KG
Potassium phosphate monobasic	Sigma	Cat# P0662-12KG
Potassium phosphate dibasic	Sigma	Cat# P3786-2.5KG
N-Z case plus	Sigma	Cat# N4642-5KG
D-Sorbitol	Sigma	Cat# S6021-5KG
D-(+)Glucose	Sigma	Cat# G8270-10KG
Protease Inhibitor Cocktail	Sigma	Cat# P8340-5ML
Restriction endonuclease DpnI	NEB	Cat# R0176S
Restriction endonuclease PmeI	NEB	Cat# R0560S
DNA polymerase	Vazyme	Cat# P515-03
Critical commercial assays		
Superdex 200 Increase 10/300 GL column	GE Healthcare	Cat# 28990944
Superose 6 Increase 10/300 GL column	GE Healthcare	Cat# 29091596
Anti-FLAG M2 Affinity Gel	Sigma	Cat# A2220-25ML
Deposited data		
Model of unbound VMAT2-del with 11 subunit CcO	This paper	PDB: 8UCJ
Model of unbound VMAT2-del with 9 subunit CcO	This paper	PDB: 8UCK
Model of VMAT2-del with tetrabenazine and 9 subunit CcO	This paper	PDB: 8UCL
Model of VMAT2-del with reserpine and 9 subunit CcO	This paper	PDB: 8UCM
Model of VMAT2-del with histamine and 9 subunit CcO	This paper	PDB: 8UCN
Model of VMAT2-del with amphetamine and 9 subunit CcO	This paper	PDB: 8UCO
Model of VMAT2-del with serotonin and 9 subunit CcO	This paper	PDB: 8UCP
Cryo-EM map of unbound VMAT2-del with 11 subunit CcO	This paper	EMD: 42128
Cryo-EM map of unbound VMAT2-del with 9 subunit CcO	This paper	EMD: 42129
Cryo-EM map of VMAT2-del with tetrabenazine and 9 subunit CcO	This paper	EMD: 42130
Cryo-EM map of VMAT2-del with reserpine and 9 subunit CcO	This paper	EMD: 42131
Cryo-EM map of VMAT2-del with histamine and 9 subunit CcO	This paper	EMD: 42132
Cryo-EM map of VMAT2-del with amphetamine and 9 subunit CcO	This paper	EMD: 42133
Cryo-EM map of VMAT2-del with serotonin and 9 subunit CcO	This paper	EMD: 42134
Experimental models: Cell lines		
SMD1163 yeast strain	Invitrogen	GenBank: XM_002489831
HEK293T cells	Sigma	Cat# 12022001
Recombinant DNA		
pBudCE4.1 empty vector	Thermo Fisher Sciencetific	Cat# V53220
pPICZ-B empty vector	Thermo Fisher Sciencetific	Cat# V19020
VMAT2 cloned into a modified pBudCE4.1 vector with FLAG tag	This paper	N/A
VMAT2 cloned into a modified pPICZ-B vector with FLAG tag	This paper	N/A
Software and algorithms		
Byonic	Protein Metrics	https://proteinmetrics.com/byonic/
Byos	Protein Metrics	https://proteinmetrics.com/byos/
Xcalibur	Thermo Fisher Scientific	https://www.thermofisher.com/order/catalog/product/OPTON-30965
Cryosparc	Punjani et al.^55^	https://cryosparc.com/
COOT	Emsley et al.^56^	http://www2.mrc-lmb.cam.ac.uk/personal/pemsley/coot
Phenix	Afonine et al.^57^	https://www.phenix-online.org/
DeepEMhancer	Sanchez-Garcia et al.^58^	https://github.com/rsanchezgarc/deepEMhancer
UCSF Chimera	Pettersen et al.^59^	https://www.cgl.ucsf.edu/chimera
ChimeraX	Pettersen et al.^60^	https://www.cgl.ucsf.edu/chimerax/
EPU	Thermo Fisher Sciencetific	https://www.thermofisher.com/us/en/home/electron-microscopy/products/software-em-3d-vis/epu-software.html
Other		
R 1.2/1.3 300 mesh Cu grid	Quantifoil	Cat# Q3100CR1.3

## Highlights

- VMAT2 stores monoamines and is targeted by neurological drugs and psychostimulants
- Cryo-EM shows VMAT2 flexibility, facilitating drug binding and monoamine storage
- Amphetamine binds the monoamine pocket but does not engage a protonation site
- Amphetamine directly induces monoamine release, likely via exchange through VMAT2

## References

[1] Carlsson A. (2001). A half-century of neurotransmitter research: impact on neurology and psychiatry. Nobel lecture. Biosci. Rep 21, 691–710. 10.1023/a:1015556204669. 12166820

[2] Backman L, Nyberg L, Lindenberger U, Li SC, and Farde L. (2006). The correlative triad among aging, dopamine, and cognition: current status and future prospects. Neurosci. Biobehav. Rev 30, 791–807. 10.1016/j.neubiorev.2006.06.005. 16901542

[3] Sara SJ (2009). The locus coeruleus and noradrenergic modulation of cognition. Nat. Rev. Neurosci 10, 211–223. 10.1038/nrn2573. 19190638

[4] Johnson RG Jr. (1988). Accumulation of biological amines into chromaffin granules: a model for hormone and neurotransmitter transport. Physiol. Rev 68, 232–307. 10.1152/physrev.1988.68.1.232. 2892215

[5] Eiden LE, Schäfer MKH, Weihe E, and Schütz B. (2004). The vesicular amine transporter family (SLC18): amine/proton antiporters required for vesicular accumulation and regulated exocytotic secretion of monoamines and acetylcholine. Pflugers Arch. 447, 636–640. 10.1007/s00424-003-1100-5. 12827358

[6] Bernstein AI, Stout KA, and Miller GW (2014). The vesicular monoamine transporter 2: an underexplored pharmacological target. Neurochem. Int 73, 89–97. 10.1016/j.neuint.2013.12.003. 24398404 PMC5028832

[7] Mawe GM, and Hoffman JM (2013). Serotonin signalling in the gut–functions, dysfunctions and therapeutic targets. Nat. Rev. Gastroenterol. Hepatol 10, 473–486. 10.1038/nrgastro.2013.105. 23797870 PMC4048923

[8] Carey RM (2001). Theodore Cooper Lecture: Renal dopamine system: paracrine regulator of sodium homeostasis and blood pressure. Hypertension 38, 297–302. 10.1161/hy0901.096422. 11566894

[9] Stone KD, Prussin C, and Metcalfe DD (2010). IgE, mast cells, baso-phils, and eosinophils. J. Allergy Clin. Immunol 125, S73–S80. 10.1016/j.jaci.2009.11.017. 20176269 PMC2847274

[10] Erickson JD, Schafer MK, Bonner TI, Eiden LE, and Weihe E. (1996). Distinct pharmacological properties and distribution in neurons and endocrine cells of two isoforms of the human vesicular monoamine transporter. Proc. Natl. Acad. Sci. USA 93, 5166–5171. 10.1073/pnas.93.10.5166. 8643547 PMC39426

[11] Akdis CA, and Blaser K. (2003). Histamine in the immune regulation of allergic inflammation. J. Allergy Clin. Immunol 112, 15–22. 10.1067/mai.2003.1585. 12847474

[12] Peter D, Vu T, and Edwards RH (1996). Chimeric vesicular monoamine transporters identify structural domains that influence substrate affinity and sensitivity to tetrabenazine. J. Biol. Chem 271, 2979–2986. 10.1074/jbc.271.6.2979. 8621690

[13] Wimalasena K. (2011). Vesicular monoamine transporters: structure-function, pharmacology, and medicinal chemistry. Med. Res. Rev 31, 483–519. 10.1002/med.20187. 20135628 PMC3019297

[14] Fei H, and Krantz DE (2009). Vesicular Neurotransmitter Transporters. In Handbook of Neurochemistry and Molecular Neurobiology: Neural Signaling Mechanisms, Lajtha A and Mikoshiba K, eds. (Springer US), pp. 87–137. 10.1007/978-0-387-30370-3_7.

[15] Efange SM (2000). In vivo imaging of the vesicular acetylcholine transporter and the vesicular monoamine transporter. FASEB J. 14, 2401–2413. 10.1096/fj.00-0204rev. 11099458

[16] Guilloteau D, and Chalon S. (2005). PET and SPECT exploration of central monoaminergic transporters for the development of new drugs and treatments in brain disorders. Curr. Pharm. Des 11, 3237–3245. 10.2174/138161205774424744. 16250852

[17] Baumeister AA (2013). The chlorpromazine enigma. J. Hist. Neurosci 22, 14–29. 10.1080/0964704X.2012.664087. 23323529

[18] Rudnick G, Steiner-Mordoch SS, Fishkes H, Stern-Bach Y, and Schuldiner S. (1990). Energetics of reserpine binding and occlusion by the chromaffin granule biogenic amine transporter. Biochemistry 29, 603–608. 10.1021/bi00455a002. 2140052

[19] Scherman D, and Henry JP (1984). Reserpine binding to bovine chromaffin granule membranes. Characterization and comparison with dihy-drotetrabenazine binding. Mol. Pharmacol 25, 113–122. 6708929

[20] Weaver JA, and Deupree JD (1982). Conditions required for reserpine binding to the catecholamine transporter on chromaffin granule ghosts. Eur. J. Pharmacol 80, 437–438. 10.1016/0014-2999(82)90093-0. 7106194

[21] Chaplin L, Cohen AH, Huettl P, Kennedy M, Njus D, and Temperley SJ (1985). Reserpic acid as an inhibitor of norepinephrine transport into chromaffin vesicle ghosts. J. Biol. Chem 260, 10981–10985. 4030777

[22] Visser SN, Danielson ML, Bitsko RH, Holbrook JR, Kogan MD, Ghandour RM, Perou R, and Blumberg SJ (2014). Trends in the parent-report of health care provider-diagnosed and medicated attention-deficit/hyperactivity disorder: United States, 2003-2011. J. Am. Acad. Child Adolesc. Psychiatry 53, 34–46.e2. 10.1016/j.jaac.2013.09.001. 24342384 PMC4473855

[23] Sulzer D, Sonders MS, Poulsen NW, and Galli A. (2005). Mechanisms of neurotransmitter release by amphetamines: a review. Prog. Neurobiol 75, 406–433. 10.1016/j.pneurobio.2005.04.003. 15955613

[24] Peter D, Jimenez J, Liu Y, Kim J, and Edwards RH (1994). The chromaffin granule and synaptic vesicle amine transporters differ in substrate recognition and sensitivity to inhibitors. J. Biol. Chem 269, 7231–7237. 8125935

[25] Schmitz Y, Lee CJ, Schmauss C, Gonon F, and Sulzer D. (2001). Amphetamine distorts stimulation-dependent dopamine overflow: effects on D2 autoreceptors, transporters, and synaptic vesicle stores. J. Neurosci 21, 5916–5924. 10.1523/JNEUROSCI.21-16-05916.2001. 11487614 PMC6763160

[26] Ing G, Hartley AM, Pinotsis N, and Maréchal A. (2022). Cryo-EM structure of a monomeric yeast S. cerevisiae complex IV isolated with maltosides: Implications in supercomplex formation. Biochim. Biophys. Acta. Bioenerg 1863, 148591. 10.1016/j.bbabio.2022.148591. 35839926

[27] Black CA, Bucher ML, Bradner JM, Jonas L, Igarza K, and Miller GW (2021). Assessing Vesicular Monoamine Transport and Toxicity Using Fluorescent False Neurotransmitters. Chem. Res. Toxicol 34, 1256–1264. 10.1021/acs.chemrestox.0c00380. 33378168 PMC8131219

[28] Hu G, Henke A, Karpowicz RJ Jr., Sonders MS, Farrimond F, Edwards R, Sulzer D, and Sames D. (2013). New fluorescent substrate enables quantitative and high-throughput examination of vesicular monoamine transporter 2 (VMAT2). ACS Chem. Biol 8, 1947–1954. 10.1021/cb400259n. 23859623 PMC4557792

[29] Wu D, Chen Q, Yu Z, Huang B, Zhao J, Wang Y, Su J, Zhou F, Yan R, Li N, (2024). Transport and inhibition mechanisms of human VMAT2. Nature 626, 427–434. 10.1038/s41586-023-06926-4. 38081299

[30] Pidathala S, Liao S, Dai Y, Li X, Long C, Chang CL, Zhang Z, and Lee CH (2023). Mechanisms of neurotransmitter transport and drug inhibition in human VMAT2. Nature 623, 1086–1092. 10.1038/s41586-023-06727-9. 37914936 PMC12832108

[31] Dalton MP, Cheng MH, Bahar I, and Coleman JA (2024). Structural mechanisms for VMAT2 inhibition by tetrabenazine. eLife 12. 10.7554/eLife.91973. PMC10959523 38517752

[32] Wang Y, Zhang P, Chao Y, Zhu Z, Yang C, Zhou Z, Li Y, Long Y, Liu Y, Li D, (2024). Transport and inhibition mechanism for VMAT2-mediated synaptic vesicle loading of monoamines. Cell Res. 34, 47–57. 10.1038/s41422-023-00906-z. 38163846 PMC10770148

[33] Yan N. (2013). Structural advances for the major facilitator superfamily (MFS) transporters. Trends Biochem. Sci 38, 151–159. 10.1016/j.tibs.2013.01.003. 23403214

[34] Quistgaard EM, Löw C, Guettou F, and Nordlund P. (2016). Understanding transport by the major facilitator superfamily (MFS): structures pave the way. Nat. Rev. Mol. Cell Biol 17, 123–132. 10.1038/nrm.2015.25. 26758938

[35] Sulzer D, and Rayport S. (1990). Amphetamine and other psychostimulants reduce pH gradients in midbrain dopaminergic neurons and chromaffin granules: a mechanism of action. Neuron 5, 797–808. 10.1016/0896-6273(90)90339-h. 2268433

[36] Yaffe D, Forrest LR, and Schuldiner S. (2018). The ins and outs of vesicular monoamine transporters. J. Gen. Physiol 150, 671–682. 10.1085/jgp.201711980. 29666153 PMC5940252

[37] Finn JP 3rd, and Edwards RH (1997). Individual residues contribute to multiple differences in ligand recognition between vesicular monoamine transporters 1 and 2. J. Biol. Chem 272, 16301–16307. 10.1074/jbc.272.26.16301. 9195934

[38] Deupree JD, and Weaver JA (1984). Identification and characterization of the catecholamine transporter in bovine chromaffin granules using [3H] reserpine. J. Biol. Chem 259, 10907–10912. 6469989

[39] Gasnier B, Krejci E, Botton D, Massoulié J, and Henry JP (1994). Expression of a bovine vesicular monoamine transporter in COS cells. FEBS Lett. 342, 225–229. 10.1016/0014-5793(94)80506-7. 8150075

[40] Parsons SM (2000). Transport mechanisms in acetylcholine and monoamine storage. FASEB J. 14, 2423–2434. 10.1096/fj.00-0203rev. 11099460

[41] Kanner BI, Fishkes H, Maron R, Sharon I, and Schuldiner S. (1979). Reserpine as a competitive and reversible inhibitor of the catecholamine transporter of bovine chromaffin granules. FEBS Lett. 100, 175–178. 10.1016/0014-5793(79)81158-8. 437101

[42] Yaffe D, Vergara-Jaque A, Forrest LR, and Schuldiner S. (2016). Emulating proton-induced conformational changes in the vesicular monoamine transporter VMAT2 by mutagenesis. Proc. Natl. Acad. Sci. USA 113, E7390–E7398. 10.1073/pnas.1605162113. 27821772 PMC5127352

[43] Schuldiner S. (2014). Competition as a way of life for H(+)-coupled antiporters. J. Mol. Biol 426, 2539–2546. 10.1016/j.jmb.2014.05.020. 24862284 PMC4072998

[44] Law CJ, Maloney PC, and Wang DN (2008). Ins and outs of major facilitator superfamily antiporters. Annu. Rev. Microbiol 62, 289–305. 10.1146/annurev.micro.61.080706.093329. 18537473 PMC2612782

[45] Khare P, Mulakaluri A, and Parsons SM (2010). Search for the acetylcholine and vesamicol binding sites in vesicular acetylcholine transporter: the region around the lumenal end of the transport channel. J. Neurochem 115, 984–993. 10.1111/j.1471-4159.2010.06990.x. 20831599 PMC2962730

[46] Pravda L, Sehnal D, Toušek D, Navrátilová V, Bazgier V, Berka K, Svobodová Vareková R, Koca J, and Otyepka M. (2018). MOLEonline: a web-based tool for analyzing channels, tunnels and pores (2018 update). Nucleic Acids Res. 46, W368–W373. 10.1093/nar/gky309. 29718451 PMC6030847

[47] Schuemann HJ, and Weigmann E. (1960). [On the point of attack of the indirect action of sympathomimetic amines]. Naunyn-Schmiedebergs Arch. Exp. Pathol. Pharmakol 240, 275–284. 13748755

[48] Shuemann HJ, and Philippu A. (1962). Release of catechol amines from isolated medullary granules by sympathomimetic amines. Nature 193, 890–891. 10.1038/193890a0. 13912355

[49] Nickell JR, Krishnamurthy S, Norrholm S, Deaciuc G, Siripurapu KB, Zheng G, Crooks PA, and Dwoskin LP (2010). Lobelane inhibits methamphetamine-evoked dopamine release via inhibition of the vesicular monoamine transporter-2. J. Pharmacol. Exp. Ther 332, 612–621. 10.1124/jpet.109.160275. 19855096 PMC2812121

[50] Dwoskin LP, and Crooks PA (2002). A novel mechanism of action and potential use for lobeline as a treatment for psychostimulant abuse. Biochem. Pharmacol 63, 89–98. 10.1016/s0006-2952(01)00899-1. 11841781

[51] Ye J, Chen H, Wang K, Wang Y, Ammerman A, Awasthi S, Xu J, Liu B, and Li W. (2024). Structural insights into vesicular monoamine storage and drug interactions. Nature 629, 235–243. 10.1038/s41586-024-07290-7. 38499039 PMC11070986

[52] Ugolev Y, Segal T, Yaffe D, Gros Y, and Schuldiner S. (2013). Identification of conformationally sensitive residues essential for inhibition of vesicular monoamine transport by the noncompetitive inhibitor tetrabenazine. J. Biol. Chem 288, 32160–32171. 10.1074/jbc.M113.502971. 24062308 PMC3820856

[53] Gros Y, and Schuldiner S. (2010). Directed evolution reveals hidden properties of VMAT, a neurotransmitter transporter. J. Biol. Chem 285, 5076–5084. 10.1074/jbc.M109.081216. 20007701 PMC2836110

[54] Finn JP 3rd, and Edwards RH (1998). Multiple residues contribute independently to differences in ligand recognition between vesicular monoamine transporters 1 and 2. J. Biol. Chem 273, 3943–3947. 10.1074/jbc.273.7.3943. 9461580

[55] Punjani A, Rubinstein JL, Fleet DJ, and Brubaker MA (2017). cryo-SPARC: algorithms for rapid unsupervised cryo-EM structure determination. Nat. Methods 14, 290–296. 10.1038/nmeth.4169. 28165473

[56] Emsley P, Lohkamp B, Scott WG, and Cowtan K. (2010). Features and development of Coot. Acta Crystallogr. D Biol. Crystallogr 66, 486–501. 10.1107/S0907444910007493. 20383002 PMC2852313

[57] Afonine PV, Grosse-Kunstleve RW, Echols N, Headd JJ, Moriarty NW, Mustyakimov M, Terwilliger TC, Urzhumtsev A, Zwart PH, and Adams PD (2012). Towards automated crystallographic structure refinement with phenix.refine. Acta Crystallogr. D Biol. Crystallogr 68, 352–367. 10.1107/S0907444912001308. 22505256 PMC3322595

[58] Sanchez-Garcia R, Gomez-Blanco J, Cuervo A, Carazo JM, Sor-zano COS, and Vargas J. (2021). DeepEMhancer: a deep learning solution for cryo-EM volume post-processing. Commun. Biol 4, 874. 10.1038/s42003-021-02399-1. 34267316 PMC8282847

[59] Pettersen EF, Goddard TD, Huang CC, Couch GS, Greenblatt DM, Meng EC, and Ferrin TE (2004). UCSF Chimera–a visualization system for exploratory research and analysis. J. Comput. Chem 25, 1605–1612. 10.1002/jcc.20084. 15264254

[60] Pettersen EF, Goddard TD, Huang CC, Meng EC, Couch GS, Croll TI, Morris JH, and Ferrin TE (2021). UCSF ChimeraX: Structure visualization for researchers, educators, and developers. Protein Sci. 30, 70–82. 10.1002/pro.3943. 32881101 PMC7737788

[61] Yaffe D, Radestock S, Shuster Y, Forrest LR, and Schuldiner S. (2013). Identification of molecular hinge points mediating alternating access in the vesicular monoamine transporter VMAT2. Proc. Natl. Acad. Sci. USA 110, E1332–E1341. 10.1073/pnas.1220497110. 23530208 PMC3625309

[62] Jumper J, Evans R, Pritzel A, Green T, Figurnov M, Ronneberger O, Tunyasuvunakool K, Bates R, Žídek A, Potapenko A, (2021). Highly accurate protein structure prediction with AlphaFold. Nature 596, 583–589. 34265844 10.1038/s41586-021-03819-2 PMC8371605

[63] Berndtsson J, Kohler A, Rathore S, Marin-Buera L, Dawitz H, Diessl J, Kohler V, Barrientos A, Büttner S, Fontanesi F, and Ott M. (2020). Respiratory supercomplexes enhance electron transport by decreasing cytochrome c diffusion distance. EMBO Rep. 21, e51015. 10.15252/embr.202051015. 33016568 PMC7726804

