# Supplementary material for: Molecular basis of vesicular monoamine transport and neurological drug interactions
Source: Cell Rep. Author manuscript; Available in PMC 2025 Dec 22. (PMC12721464; doi:10.1016/j.celrep.2025.116490)
Supplement: 1 [file NIHMS2125813-supplement-1.pdf]

Cell Reports, Volume *44*

Supplemental information

Molecular basis of vesicular monoamine transport and neurological drug interactions

Jin Ye, Huaping Chen, Aaron Ammerman, Yi Wang, Kaituo Wang, Jinbin Xu, Bin Liu, and Weikai Li

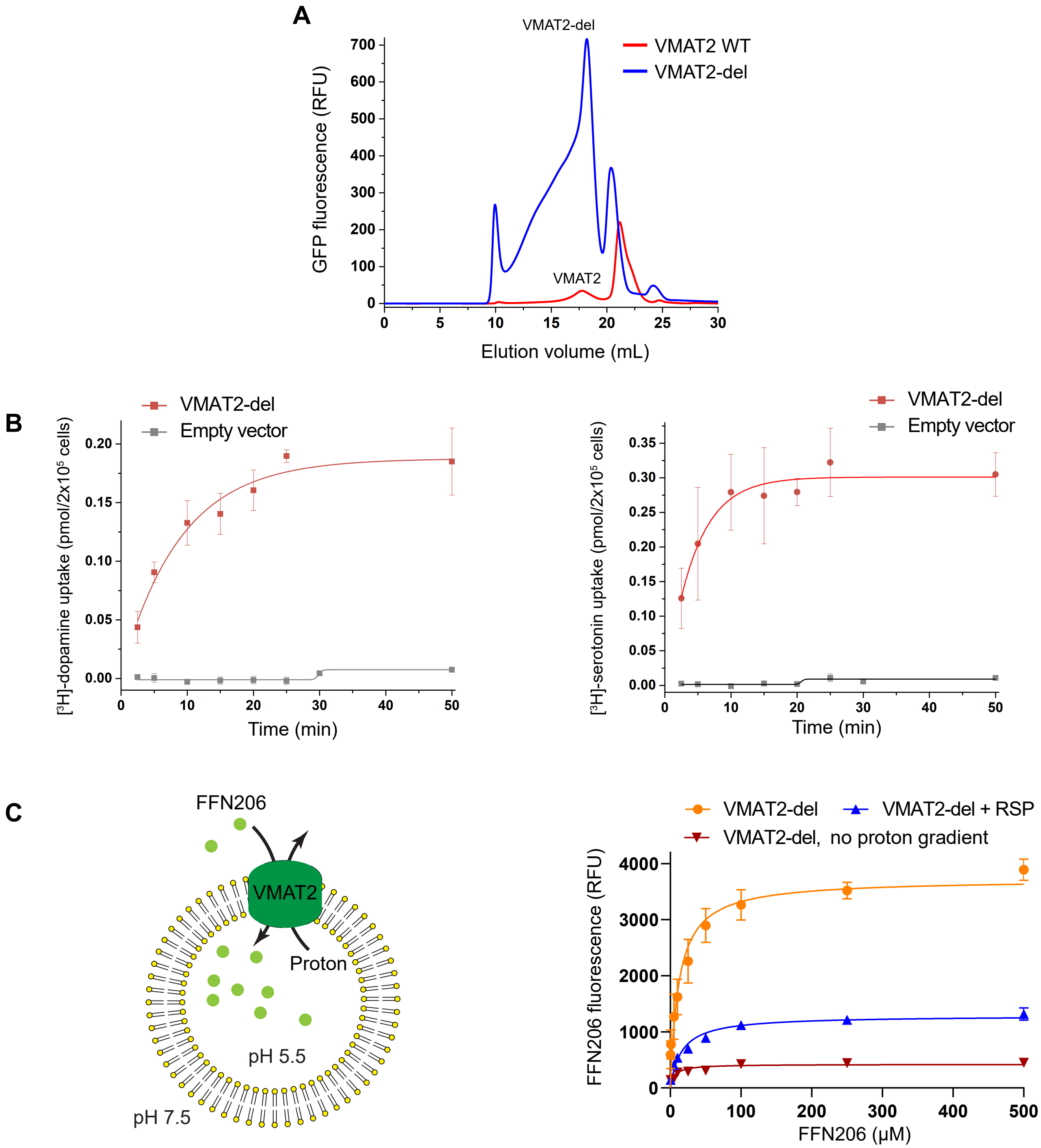

Figure S1. Enhanced protein yield and active monoamine uptake by the VMAT2-del construct, related to Figure 1.

(A) FSEC profile comparison of full-length human VMAT2 and VMAT2-del. These proteins are tagged with a C-terminal GFP, expressed in *Pichia pastoris* cells, and extracted in DDM.

(B) Time course of [^{3}H]-dopamine (*left*) and [^{3}H]-serotonin (*right*) uptake mediated by VMAT2-del. Based on the linear range of these curves, the uptake assays were performed 25 min for [^{3}H]-dopamine and 10 min for [^{3}H]-serotonin. Errors are s.e.m. from three repeats.

(C) FFN206 transport into liposomes by purified VMAT2-del protein expressed in *Pichia pastoris*. *Left*, Scheme of the liposome assay. *Right*, Saturation curve of FFN206 transport. Transport dependents on the proton gradient and is inhibited by reserpine. Errors are s.e.m. from three repeats.

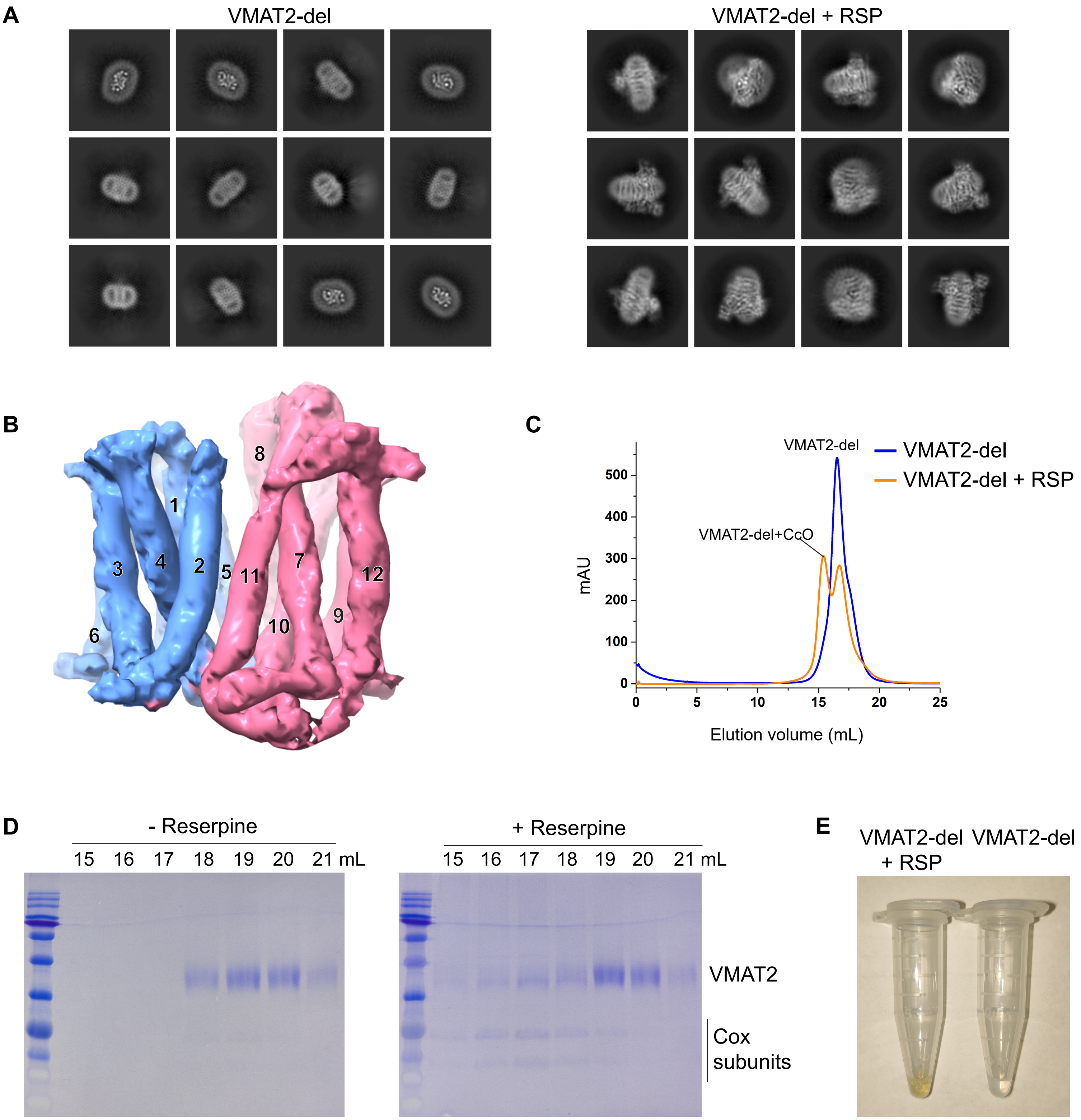

Figure S2. Formation of VMAT2-CcO complex induced by reserpine treatment during protein purification, related to Figure 1.

(A) 2D classification showing that VMAT2 protein forms monomers without reserpine treatment (*left*) and forms a large complex with reserpine treatment (*right*). The particles were extracted at the same box size of 320 pixels (0.664 Å/pixel).

(B) Low-resolution map (contoured at 0.4 in ChimeraX) of the VMAT2-del monomer.

(C) Size-exclusion chromatography (Superose 6) elution profile showing a higher molecular weight shift after reserpine treatment, suggestive of complex formation.

(D) SDS-PAGE of eluted fractions from the size-exclusion chromatography.

(E) The purified, reserpine-pretreated protein exhibits a yellow color.

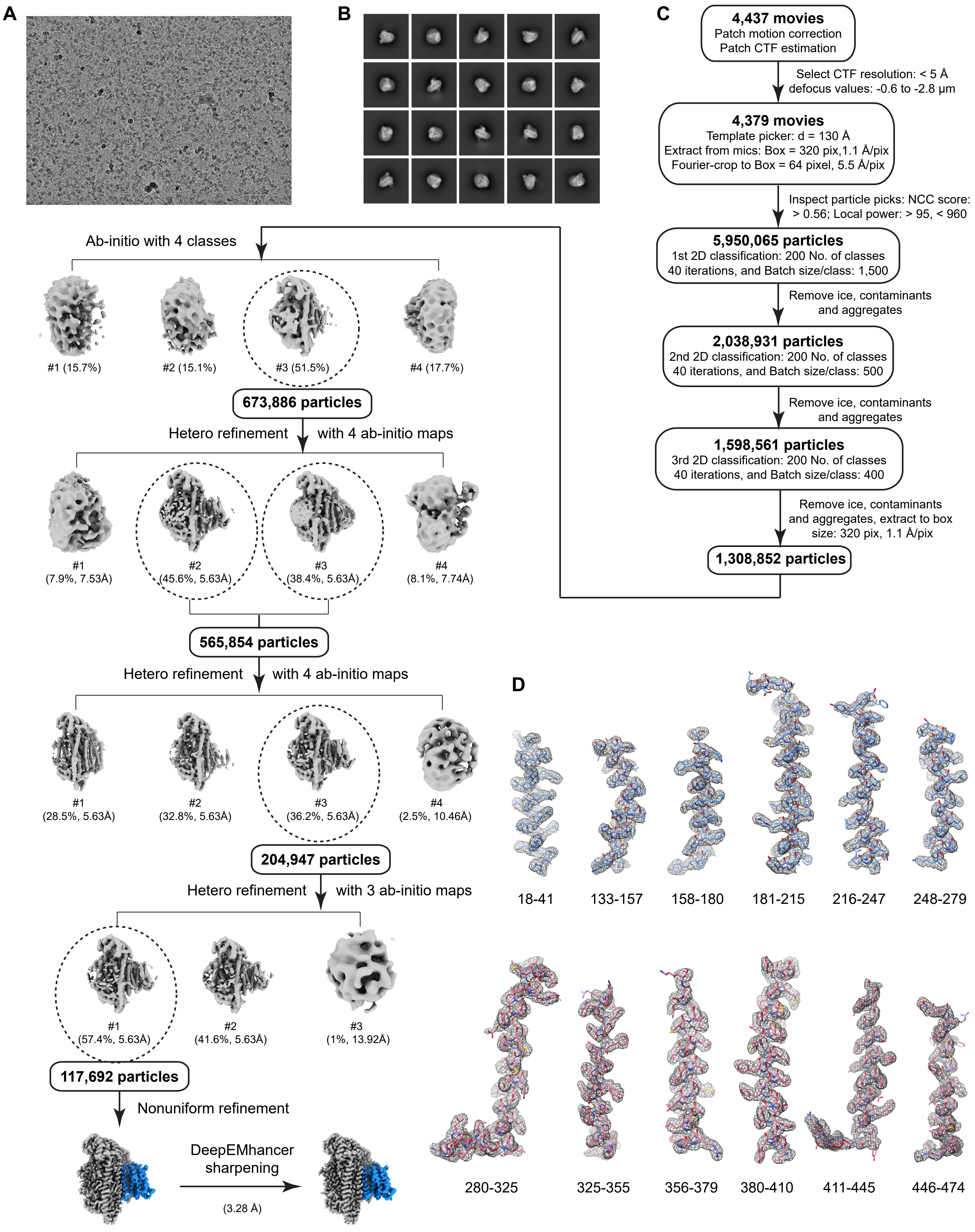

A
B
C
4,437 movies
Patch motion correction
Patch CTF estimation
Select CTF resolution: < 5 Å
defocus values: -0.6 to -2.8 µm
4,379 movies
Template picker: d = 130 Å
Extract from mics: Box = 320 pix,1.1 Å/pix
Fourier-crop to Box = 64 pixel, 5.5 Å/pix
Inspect particle picks: NCC score: > 0.56; Local power: > 95, < 960
5,950,065 particles
1st 2D classification: 200 No. of classes
40 iterations, and Batch size/class: 1,500
Remove ice, contaminants and aggregates
2,038,931 particles
2nd 2D classification: 200 No. of classes
40 iterations, and Batch size/class: 500
Remove ice, contaminants and aggregates
1,598,561 particles
3rd 2D classification: 200 No. of classes
40 iterations, and Batch size/class: 400
Remove ice, contaminants and aggregates, extract to box size: 320 pix, 1.1 Å/pix
1,308,852 particles
Ab-initio with 4 classes
#1 (15.7%)
#2 (15.1%)
#3 (51.5%)
#4 (17.7%)
673,886 particles
Hetero refinement with 4 ab-initio maps
#1 (7.9%, 7.53Å)
#2 (45.6%, 5.63Å)
#3 (38.4%, 5.63Å)
#4 (8.1%, 7.74Å)
565,854 particles
Hetero refinement with 4 ab-initio maps
#1 (28.5%, 5.63Å)
#2 (32.8%, 5.63Å)
#3 (36.2%, 5.63Å)
#4 (2.5%, 10.46Å)
204,947 particles
Hetero refinement with 3 ab-initio maps
#1 (57.4%, 5.63Å)
#2 (41.6%, 5.63Å)
#3 (1%, 13.92Å)
117,692 particles
Nonuniform refinement
DeepEMhancer sharpening
(3.28 Å)
D
18-41
133-157
158-180
181-215
216-247
248-279
280-325
325-355
356-379
380-410
411-445
446-474

Figure S3. Cryo-EM data processing flowchart, related to Figure 1.

The dataset processing of VMAT2-del in unbound state is shown as a representative, and other datasets are similarly processed.

(A) A representative raw cryo-EM image.

(B) Representative 2D classes.

(C) The data processing procedure. Four rounds of 2D classification and four rounds of heterogenous refinements generated 3.28 Å final maps. The maps were sharpened by DeepEMhancer for model building and analysis.

(D) Density maps of individual regions.

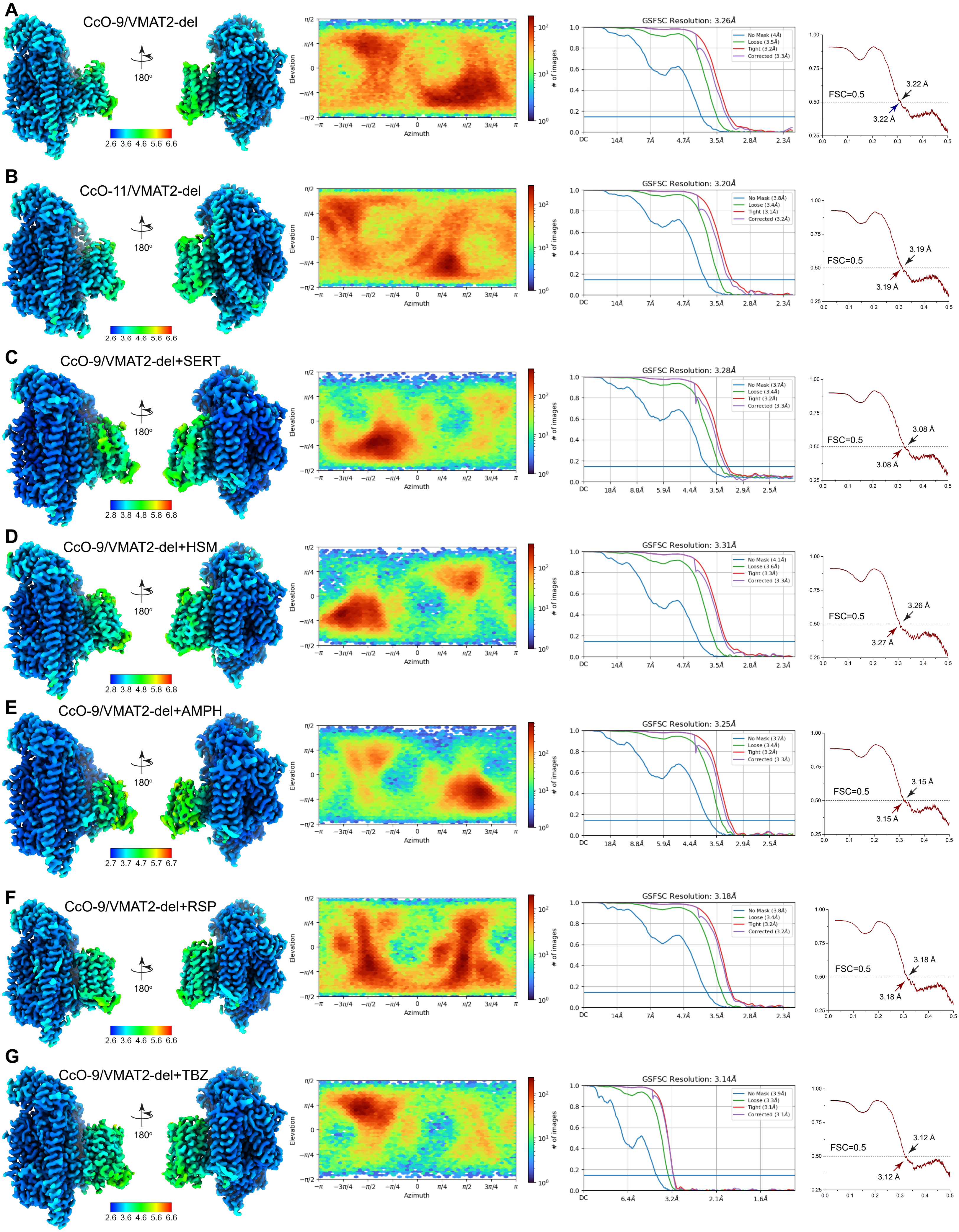
A
CcO-9/VMAT2-del
180°
2.6 3.6 4.6 5.6 6.6
GSFSC Resolution: 3.26Å
No Mask (4Å)
Loose (3.5Å)
Tight (3.2Å)
Corrected (3.3Å)
FSC=0.5
3.22 Å
3.22 Å
B
CcO-11/VMAT2-del
180°
2.6 3.6 4.6 5.6 6.6
GSFSC Resolution: 3.20Å
No Mask (3.8Å)
Loose (3.4Å)
Tight (3.1Å)
Corrected (3.2Å)
FSC=0.5
3.19 Å
3.19 Å
C
CcO-9/VMAT2-del+SERT
180°
2.8 3.8 4.8 5.8 6.8
GSFSC Resolution: 3.28Å
No Mask (3.7Å)
Loose (3.4Å)
Tight (3.2Å)
Corrected (3.3Å)
FSC=0.5
3.08 Å
3.08 Å
D
CcO-9/VMAT2-del+HSM
180°
2.8 3.8 4.8 5.8 6.8
GSFSC Resolution: 3.31Å
No Mask (4.1Å)
Loose (3.6Å)
Tight (3.3Å)
Corrected (3.3Å)
FSC=0.5
3.26 Å
3.27 Å
E
CcO-9/VMAT2-del+AMPH
180°
2.7 3.7 4.7 5.7 6.7
GSFSC Resolution: 3.25Å
No Mask (3.7Å)
Loose (3.4Å)
Tight (3.2Å)
Corrected (3.3Å)
FSC=0.5
3.15 Å
3.15 Å
F
CcO-9/VMAT2-del+RSP
180°
2.6 3.6 4.6 5.6 6.6
GSFSC Resolution: 3.18Å
No Mask (3.8Å)
Loose (3.4Å)
Tight (3.2Å)
Corrected (3.2Å)
FSC=0.5
3.18 Å
3.18 Å
G
CcO-9/VMAT2-del+TBZ
180°
2.6 3.6 4.6 5.6 6.6
GSFSC Resolution: 3.14Å
No Mask (3.9Å)
Loose (3.3Å)
Tight (3.1Å)
Corrected (3.1Å)
FSC=0.5
3.12 Å
3.12 Å
Elevation
Azimuth
# of images

Figure S4. Quality of cryo-EM maps, related to Figure 1.

(A) The complex of CcO and VMAT2-del. The CcO is in the 9-subunit form (Cco-9) and VMAT2-del (CcO-VMAT2) in unbound form. *Left to right,* Local resolution illustrations of the cryo-EM maps, angular distribution plots, half-map FSC curves, and model-to-map FSCs (unmasked). The local resolution of VMAT2-del is 3.0-4.6 Å.

(B) Respective representations for complexes of CcO in 11-subunit form (Cco-11) and VMAT2-del in unbound form. The local resolution of VMAT2-del is 3.0-4.6 Å.

(C) Respective representations for CcO-VMAT2 with serotonin (SERT). The local resolution of VMAT2-del is 3.6-5.0 Å, and around the serotonin binding site is 3.6-4.3 Å.

(D) CcO-VMAT2 with histamine (HSM). The local resolution of VMAT2-del is 3.6-4.8 Å, and around the histamine binding site is 3.6-4.2 Å.

(E) CcO-VMAT2 with amphetamine (AMPH). The local resolution of VMAT2-del is 3.7-5.2 Å, and around the amphetamine binding site is 3.7-4.5 Å.

(F) CcO-VMAT2 with reserpine (RSP). The local resolution of VMAT2-del is 3.2-4.6 Å, and around the reserpine binding site is 3.2-3.8 Å.

(G) CcO-VMAT2 with tetrabenazine (TBZ). The local resolution of VMAT2-del is 3.2-4.6 Å, and around the tetrabenazine binding site is 3.2-3.6 Å.

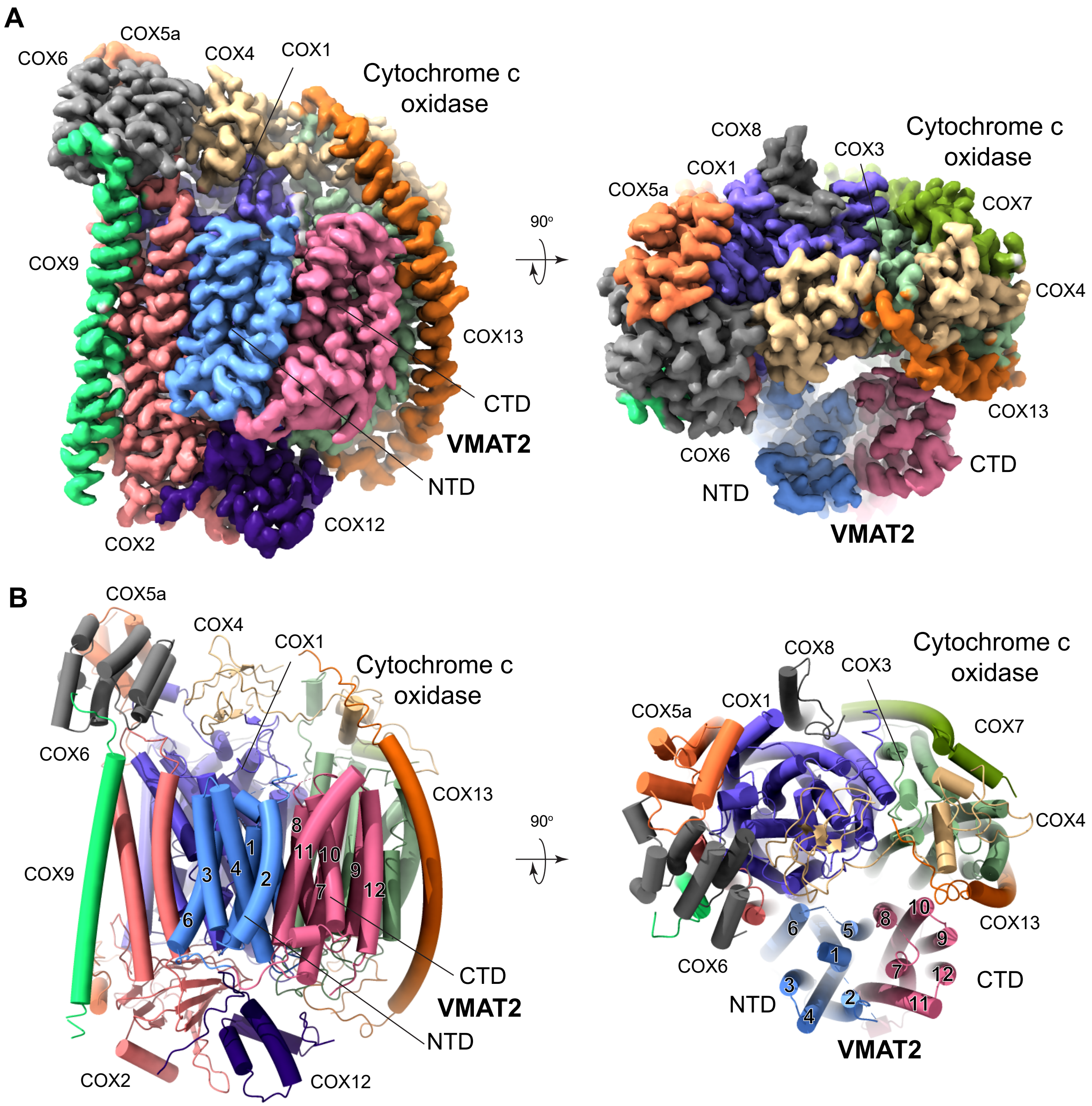

Figure S5. Overall structure of the VMAT2-CcO complex, related to Figure 1.

(A) Cryo-EM density map (contour level = 0.03 in ChimeraX) of the VMAT2-CcO complex shown in side view (left) and top view (right). The NTD of VMAT2 is shown in blue, CTD in red, and CcO subunits (COX1-13) in distinct colors.

(B) Structure of the VMAT2-CcO complex in the unbound state, displayed in the same color and orientation as in (A). VMAT2 associates with COX1-3, the core complex of CcO. The numbers indicate transmembrane helices in VMAT2.

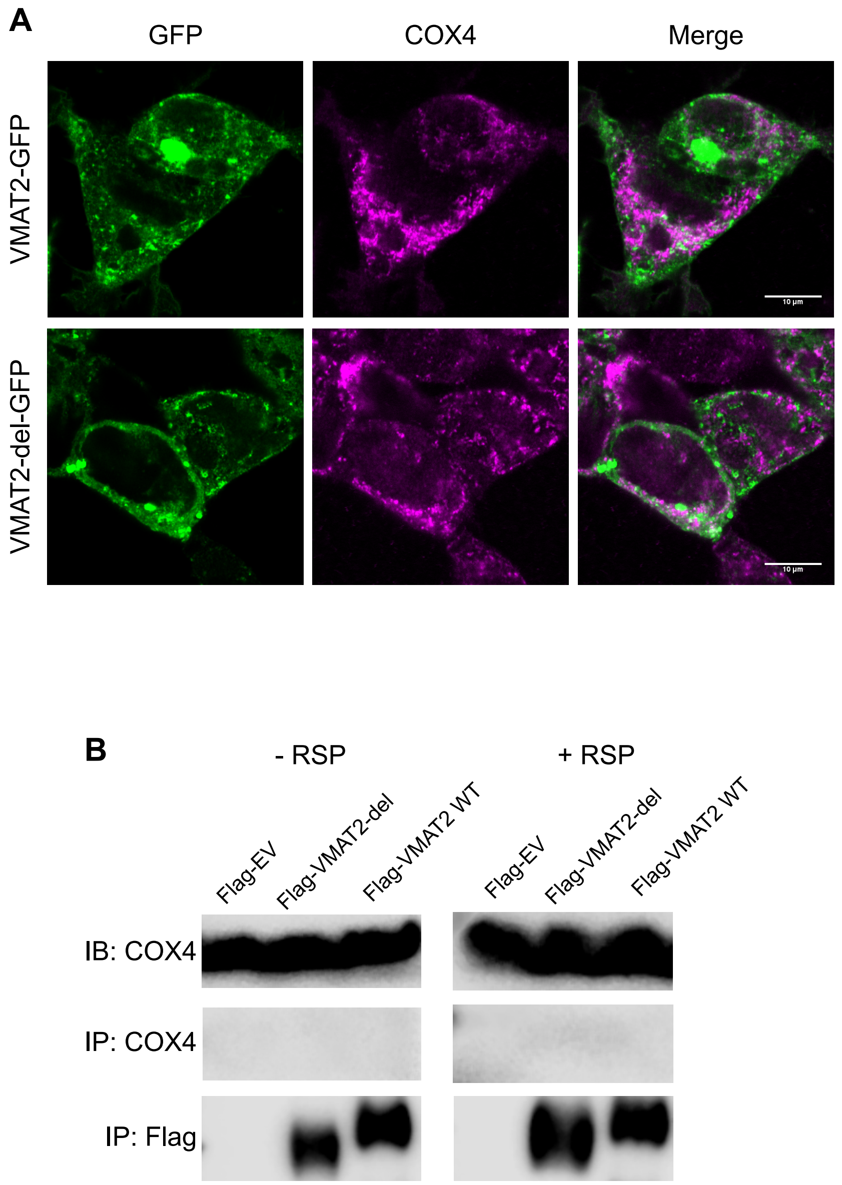

Figure S6. Exploring the physiological significance of VMAT2 and CcO complex formation, related to Figure 1.

(A) Confocal microscopy imaging reveals regions of partial colocalization between VMAT2 and CcO within HEK293 cells.

(B) Co-immunoprecipitation (Co-IP) fails to detect the complex formation. The Co-IP experiment was conducted with triton X-100, and the use of detergents may affect the complex formation in vitro.

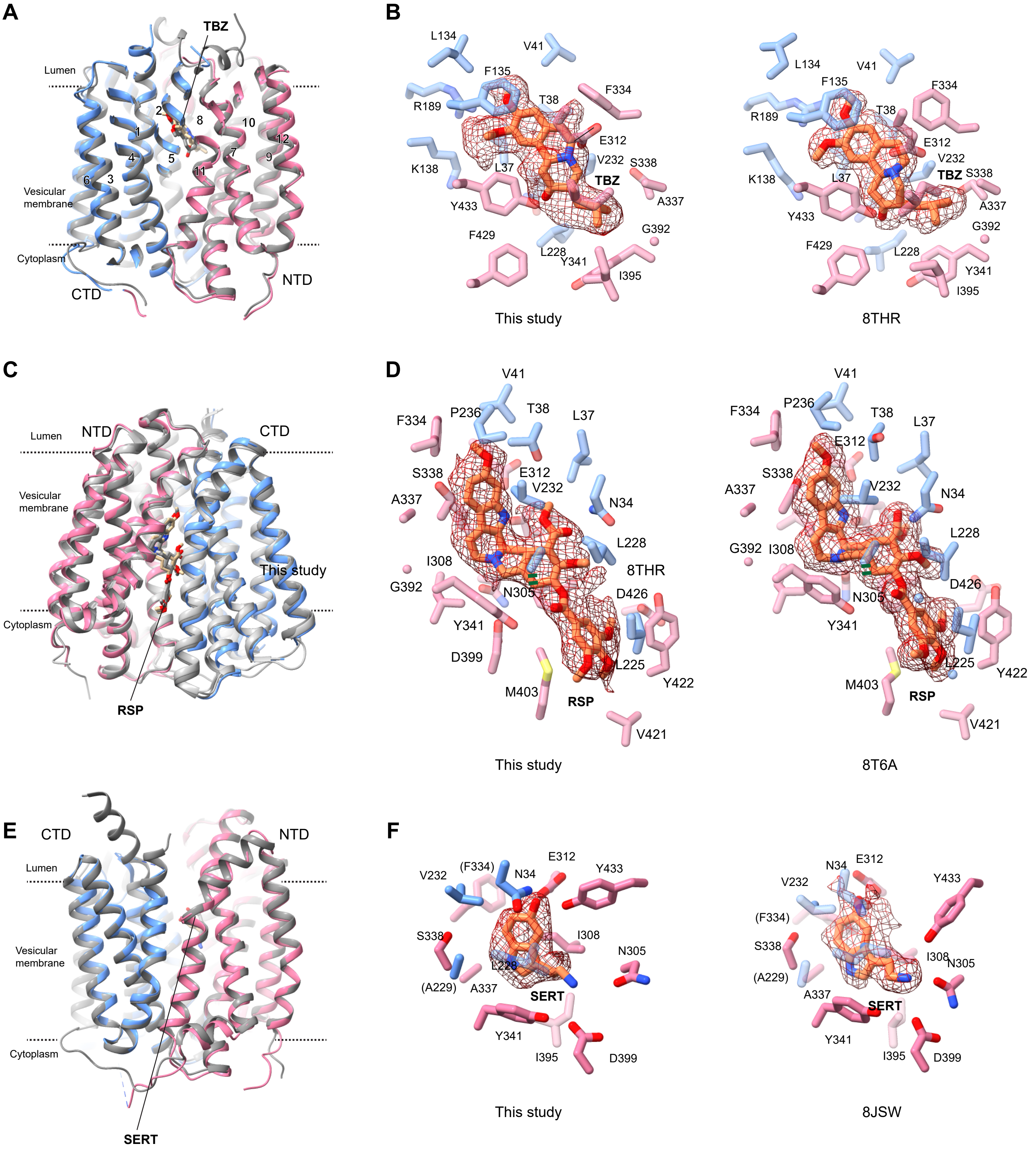

Figure S7. Comparison with reported structures, related to Figure 1.

(A) Superimposition of the tetrabenazine-bound structure from this study (NTD in red, CTD in blue) with the published structure 8THR from [S1] (grey).

(B) Tetrabenazine binding site. Left: this study. Map contour = 0.03 in ChimeraX. Right: 8THR. Map contour = 0.12.

(C) Superimposition of the reserpine-bound structure from this study with 8T6A [S2].

(D) The reserpine binding site. Left: this study. Map contour = 0.11. Right: 8T6A. Map contour = 0.03.

(E) Superimposition of the serotonin-bound structure from this study with 8JSW [S3].

(F) Serotonin binding site. Left: this study. Map contour = 0.003. Right: 8JSW. Map contour = 0.4.

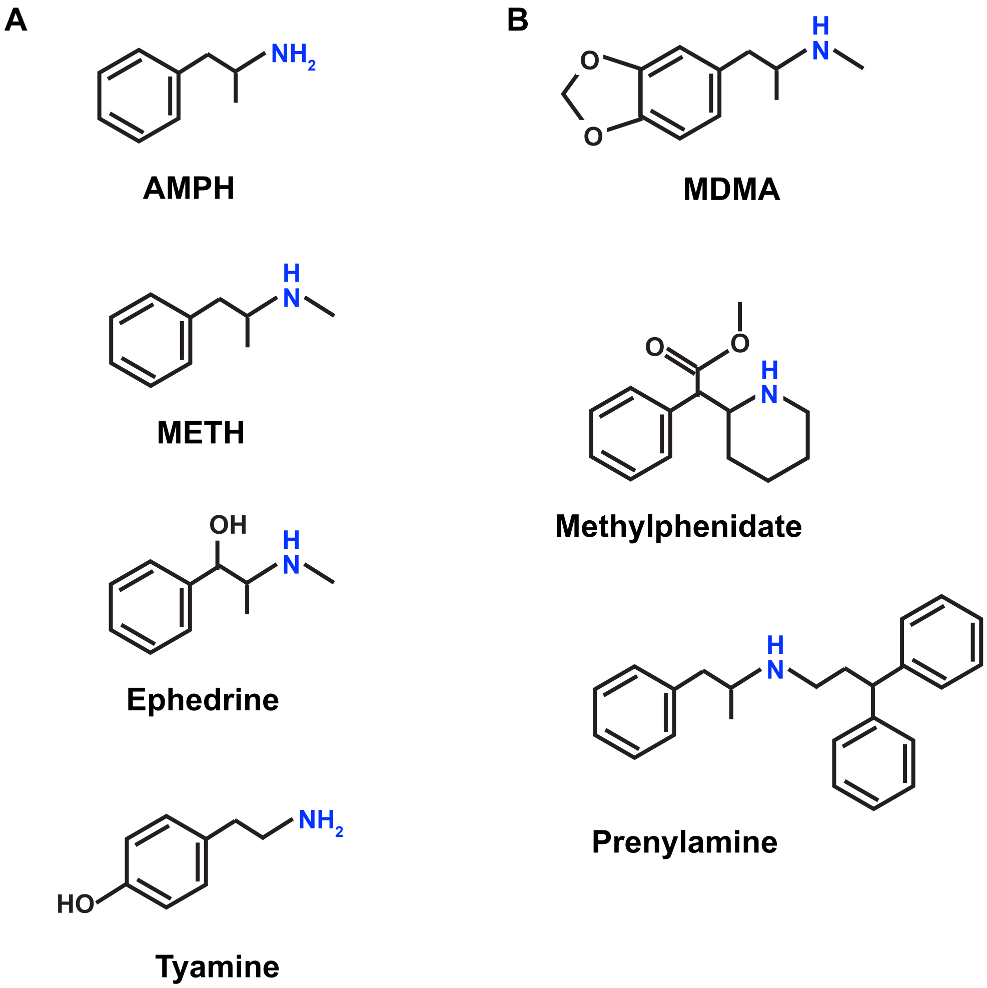

Figure S8. Chemical structures of representative amphetamine-related illicit and clinical drugs, related to Figure 4.

(A) Compounds with minor modifications to amphetamine. Amphetamine (AMPH) and methamphetamine (METH) and are traditionally abused substances. Ephedrine, used clinically to treat significant hypotension, also serves as a precursor in METH synthesis. Tyramine, a non-α-methylated phenethylamine, is a paradigmatic false transmitter often with microbial origins, found in red wine, beer, and cheese.

(B) Compounds with moderate modifications to amphetamine. 3,4-Methylenedioxymethamphetamine (MDMA: ecstasy) and methylphenidate (Ritalin) represent ring-substituted amphetamines. Prenylamine represents chain-substituted amphetamines.

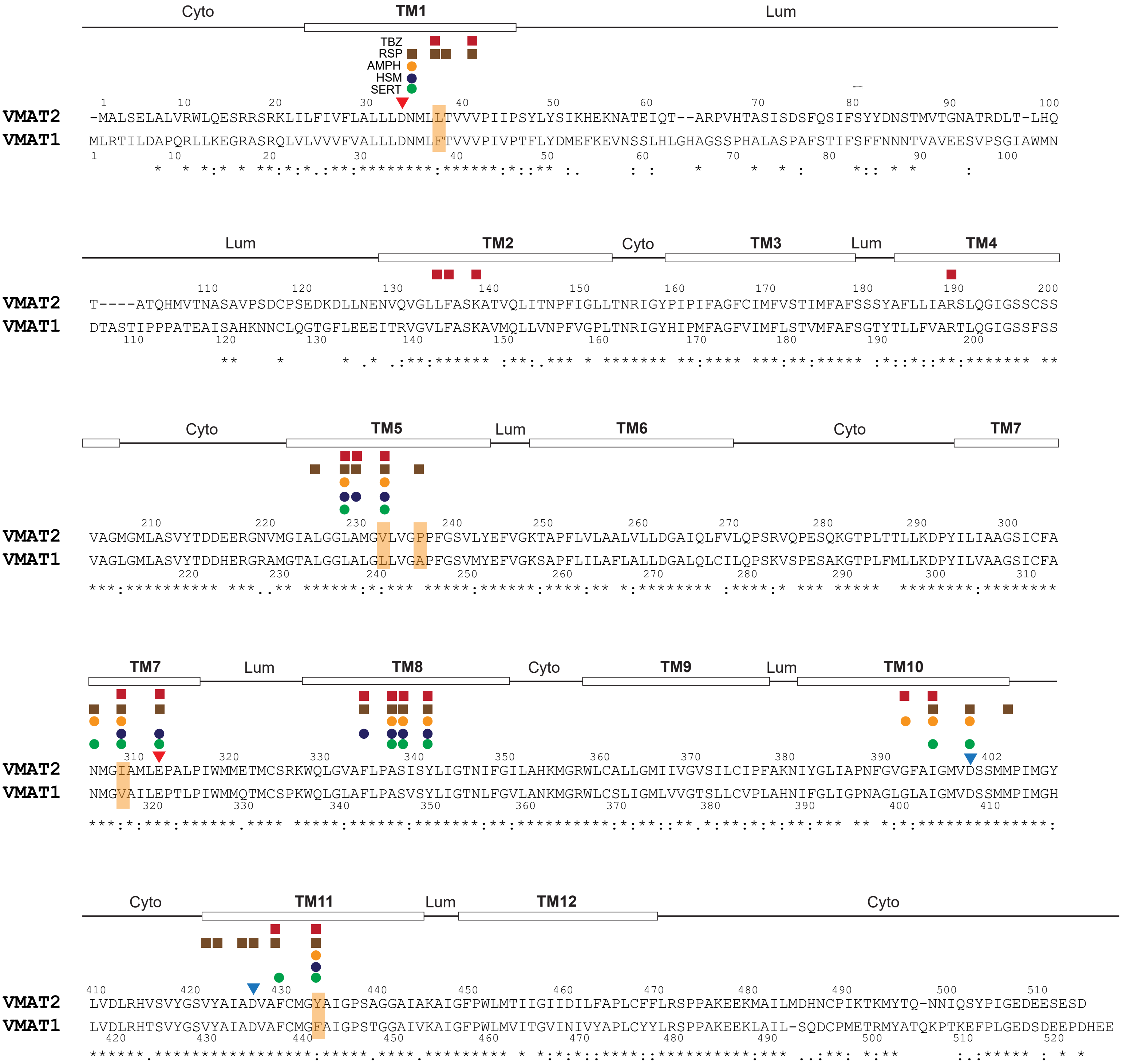

Figure S9. Sequence alignment of human VMAT1 and VMAT2, related to Figure 5.

The sequence alignment was generated by ClustalW. The asterisks below the aligned sequences indicate conserved residues, and single and double dots represent varying levels of sequence similarity. Residues participating in serotonin, histamine, amphetamine, reserpine, and tetrabenazine binding are highlighted with green, dark blue, orange, brown, and red spheres or squares above the sequences, respectively. The orange shading indicates key binding residues that are different in VMAT2 and VMAT1. The potential protonation sites, D33 and E320, are indicated by red triangles. Other protonatable sites inside TMs are indicated by blue triangles. The protein folding topology (Cyto: cytoplasmic side. Lum: lumenal side), based on the VMAT2 structure, is denoted above the sequences. The TM boundaries are calculated by PPM 3.0 web server [S4].

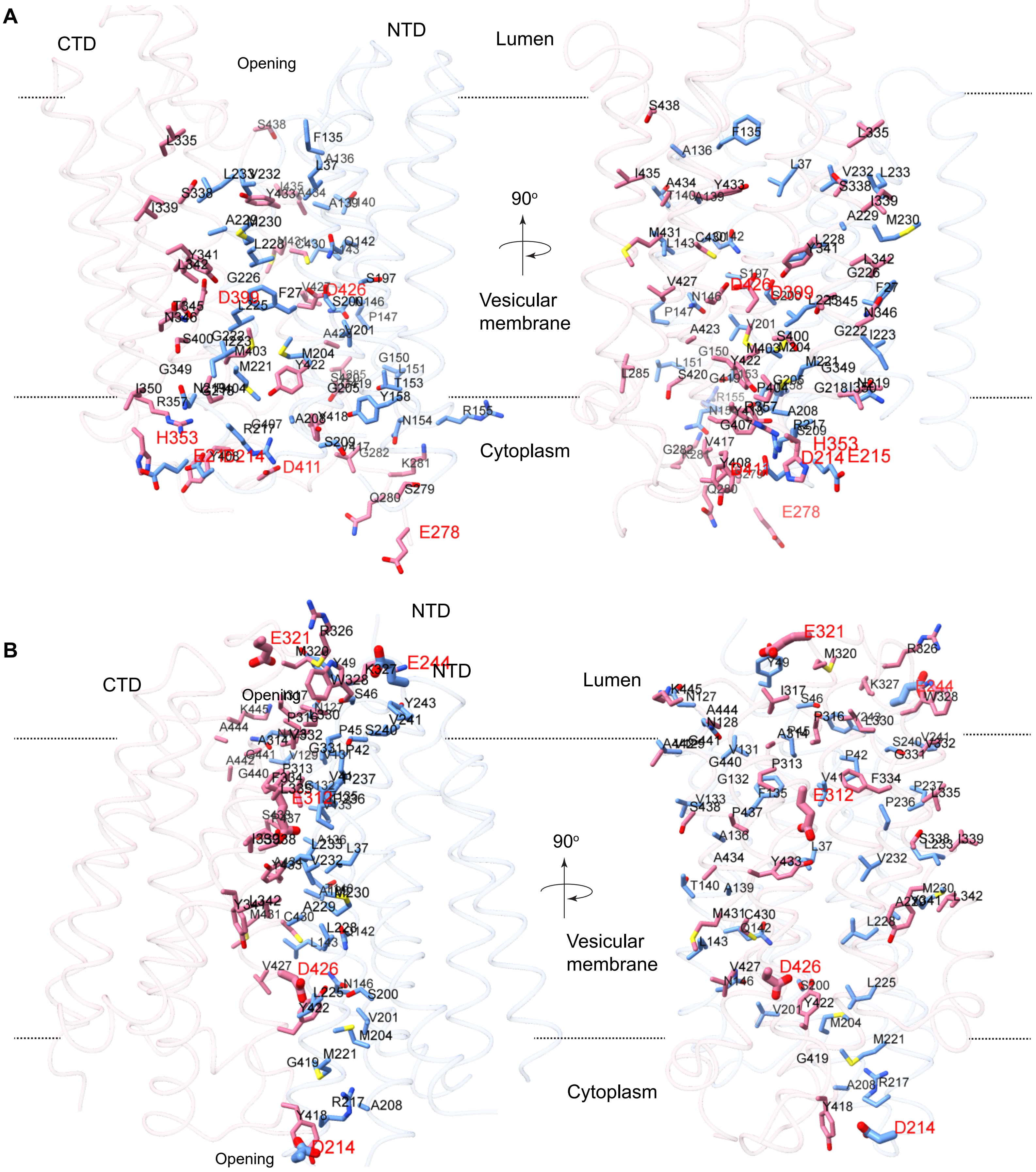

Figure S10. Gating interactions in vesicular lumenal-open and cytoplasmic-open conformations, related to Figure 7.

(A) Gating residues specific to the lumenal-open conformation when serotonin is bound. Interaction analysis (within a 4.5 Å distance) was conducted using the CONTACT command in ChimeraX. Acidic residues and histidines are highlighted with red labels.

(B) Gating residues specific to the cytoplasmic-open conformation when reserpine is bound.

Table S1. Cryo-EM data collection, refinement, and validation statistics

	CcO-VMAT2 (11 subunits) (EMD-42128) (PDB 8UCJ)	CcO-VMAT2 (9 subunits) (EMD-42129) (PDB 8UCK)	CcO-VMAT2-TBZ (EMD-42130) (PDB 8UCL)	CcO-VMAT2-RSP (EMD-42131) (PDB 8UCM)
**Data collection and processing**				
Magnification	81,000	81,000	81,000	130,000
Voltage (kV)	300	300	300	300
Electron exposure (e–/Å^2)	50	50	50	50
Defocus range (μm)	−1.0 ~ −2.2	−1.0 ~ −2.0	−1.0 ~ −2.0	−1.0 ~ −2.0
Pixel size (Å)	1.1	1.1	1.1	0.664
Symmetry imposed	C1	C1	C1	C1
Initial particle images (no.)	444,171	444,171	908,822	854,507
Final particle images (no.)	141,043	105,062	116,216	61,624
Map resolution (Å)	3.20	3.26	3.18	3.14
FSC threshold	0.143	0.143	0.143	0.143
Map resolution range (Å)	2.6-6.6	2.6-6.6	2.6-6.6	2.6-6.6
**Refinement**				
Model resolution (Å)	3.19	3.22	3.18	3.12
FSC threshold	0.5	0.5	0.5	0.5
Map sharpening *B* factor (Å^2)	111.1	100.6	103.8	82.0
Model composition				
Non-hydrogen atoms	16,794	15,148	15,439	15,470
Protein residues	2,081	1,887	1,916	1,916
Ligands	10	9	10	10
B factors (Å^2)				
Protein	66.88	63.76	59.96	54.58
Ligand	62.59	57.23	58.31	46.67
R.m.s. deviations				
Bond lengths (Å)	0.006	0.007	0.873	0.951
Bond angles (°)	1.161	1.171	0.005	0.007
Validation				
MolProbity score	1.42	1.41	2.04	2.11
Clashscore	2.35	2.20	14.09	16.12
Poor rotamers (%)	0.00	0.00	0.00	0.00
Ramachandran plot				
Favored (%)	93.86	93.71	94.29	94.08
Allowed (%)	6.05	6.18	5.66	5.87
Disallowed (%)	0.10	0.11	0.05	0.05

Table S1. Continued.

	CcO-VMAT2-HSM (EMD-42132) (PDB 8UCN)	CcO-VMAT2-AMPH (EMD-42133) (PDB 8UCO)	CcO-VMAT2-SERT (EMD-42134) (PDB 8UCP)
**Data collection and processing**			
Magnification	81,000	81,000	81,000
Voltage (kV)	300	300	300
Electron exposure (e–/Å^2)	50	50	50
Defocus range (μm)	−1.0 ~ −2.0	−1.0 ~ −2.0	−1.0 ~ −2.0
Pixel size (Å)	1.1	1.1	1.1
Symmetry imposed	C1	C1	C1
Initial particle images (no.)	1,224,600	1,081,596	1,308,852
Final particle images (no.)	84,534	114,678	117,692
Map resolution (Å)	3.31	3.25	3.28
FSC threshold	0.143	0.143	0.143
Map resolution range (Å)	2.8-6.8	2.7-6.7	2.8-6.8
**Refinement**			
Model resolution (Å)	3.26	3.15	3.08
FSC threshold	0.5	0.5	0.5
Map sharpening *B* factor (Å^2)	102.3	97.4	102.0
Model composition			
Non-hydrogen atoms	15,312	15,134	15,361
Protein residues	1,905	1,877	1,905
Ligands	10	11	11
B factors (Å^2)			
Protein	61.21	61.69	51.61
Ligand	55.80	52.84	43.46
R.m.s. deviations			
Bond lengths (Å)	0.005	0.006	0.007
Bond angles (°)	0.870	0.872	0.907
Validation			
MolProbity score	2.09	2.15	2.14
Clashscore	16.01	17.62	17.90
Poor rotamers (%)	0.00	0.00	0.00
Ramachandran plot			
Favored (%)	94.42	93.94	94.31
Allowed (%)	5.53	6.00	5.64
Disallowed (%)	0.05	0.05	0.05

SUPPLEMENTAL REFERENCES

S1. Dalton, M.P., Cheng, M.H., Bahar, I., and Coleman, J.A. (2024). Structural mechanisms for VMAT2 inhibition by tetrabenazine. Elife *12*. 10.7554/eLife.91973.

S2. Pidathala, S., Liao, S., Dai, Y., Li, X., Long, C., Chang, C.L., Zhang, Z., and Lee, C.H. (2023). Mechanisms of neurotransmitter transport and drug inhibition in human VMAT2. Nature *623*, 1086-1092. 10.1038/s41586-023-06727-9.

S3. Wu, D., Chen, Q., Yu, Z., Huang, B., Zhao, J., Wang, Y., Su, J., Zhou, F., Yan, R., Li, N., et al. (2024). Transport and inhibition mechanisms of human VMAT2. Nature *626*, 427-434. 10.1038/s41586-023-06926-4.

S4. Lomize, A.L., Todd, S.C., and Pogozheva, I.D. (2022). Spatial arrangement of proteins in planar and curved membranes by PPM 3.0. Protein Sci *31*, 209-220. 10.1002/pro.4219.
